# Molecular and functional characterization of the SBP-box transcription factor SPL-CNR in tomato fruit ripening and cell death

**DOI:** 10.1093/jxb/eraa067

**Published:** 2020-02-04

**Authors:** Tongfei Lai, Xiaohong Wang, Bishun Ye, Mingfei Jin, Weiwei Chen, Ying Wang, Yingying Zhou, Andrew M Blanks, Mei Gu, Pengcheng Zhang, Xinlian Zhang, Chunyang Li, Huizhong Wang, Yule Liu, Philippe Gallusci, Mahmut Tör, Yiguo Hong

**Affiliations:** 1 Research Centre for Plant RNA Signaling and Zhejiang Provincial Key Laboratory for Genetic Improvement and Quality Control of Medicinal Plants, College of Life and Environmental Sciences, Hangzhou Normal University, Hangzhou, China; 2 School of Life Sciences, East China Normal University, Shanghai, China; 3 Warwick-Hangzhou Joint RNA Signaling Laboratory, School of Life Sciences, University of Warwick, Coventry, UK; 4 Cell and Developmental Biology, Division of Biomedical Sciences, Warwick Medical School, University of Warwick, Coventry, UK; 5 The Gurdon Institute, University of Cambridge, Cambridge, UK; 6 Department of Family Medicine and Public Health, Division of Biostatistics & Bioinformatics, University of California San Diego, La Jolla, CA, USA; 7 MOE Key Laboratory of Bioinformatics, Centre for Plant Biology, School of Life Sciences, Tsinghua University, Beijing, China; 8 Laboratory of Grape Ecophysiology and Functional Biology, Bordeaux University, INRA, Bordeaux Science Agro, Villenave d’Ornon, France; 9 Worcester-Hangzhou Joint Molecular Plant Health Laboratory, School of Science and the Environment, University of Worcester, Worcester, UK; 10 Fondazione Edmund Mach, Italy

**Keywords:** Cell death, *Colourless non-ripening*, nuclear localization signal, SlSnRK1, SlSPL-CNR, tomato (*Solanum lycopersicum*) fruit ripening, zinc-finger motif

## Abstract

*SlSPL-CNR*, an SBP-box transcription factor (TF) gene residing at the epimutant *Colourless non-ripening* (*Cnr*) locus, is involved in tomato ripening. This epimutant provides a unique model to investigate the (epi)genetic basis of fruit ripening. Here we report that SlSPL-CNR is a nucleus-localized protein with a distinct monopartite nuclear localization signal (NLS). It consists of four consecutive residues ‘ _30_KRKR_33_’ at the N-terminus of the protein. Mutation of the NLS abolishes SlSPL-CNR’s ability to localize in the nucleus. SlSPL-CNR comprises two zinc-finger motifs (ZFMs) within the C-terminal SBP-box domain. Both ZFMs contribute to zinc-binding activity. SlSPL-CNR can induce cell death in tomato and tobacco, dependent on its nuclear localization. However, the two ZFMs have differential impacts on SlSPL-CNR’s induction of severe necrosis or mild necrotic ringspot. NLS and ZFM mutants cannot complement *Cnr* fruits to ripen. SlSPL-CNR interacts with SlSnRK1. Virus-induced *SlSnRK1* silencing leads to reduction in expression of ripening-related genes and inhibits ripening in tomato. We conclude that SlSPL-CNR is a multifunctional protein that consists of a distinct monopartite NLS, binds to zinc, and interacts with SlSnRK1 to affect cell death and tomato fruit ripening.

## Teaser

The tomato transcription factor SlSPL-CNR is a multi-functional protein that has a role in fruit ripening and is capable of triggering cell death.

## Introduction

*Cnr* is a naturally occurring epimutant of tomato. *Cnr* plants undergo normal growth and development, but fruits cannot ripen and remain colourless. The texture of *Cnr* tomato is altered due to a loss of cell-to-cell adhesion in the fruit tissues (Eriksson *et al.*, 2004). Mapping and positional cloning reveal that the *Cnr* locus harbours an SBP-box gene, *CNR* (*LeSPL-CNR*, redesigned as *SlSPL-CNR*), belonging to the SPL gene family of transcription factors (TFs; Manning *et al.*, 2006; Kong *et al.*, 2013; Chen *et al.*, 2018*a*). This mutant results from a spontaneous epimutation that causes hypermethylation in the 286 bp DNA region of the promoter, approximately 2.4 kb upstream of the *SlSPL-CNR* gene coding sequence. *SlSPL-CNR* is developmentally regulated, being mainly expressed in ripening fruits (Manning *et al.*, 2006; Salinas *et al.*, 2012), with its expression fine-tuned by *SlymiR157* (*SlmiR157*) to affect fruit ripening (Chen *et al.*, 2015*a*). *Cnr* has a hypermethylated epigenome (Zhong *et al.*, 2013; Chen *et al.*, 2015*b*), likely due to lack of expression of *SlDML2* (Liu *et al.*, 2015). *SlCMT2*, *SlCMT3*, *SlDRM7*, and *SlMET1*, which are key genes in the RNA-directed DNA methylation and methylation maintenance pathways, are required to maintain the *Cnr* epiallele. Inhibition of these genes by virus-induced gene silencing (VIGS) results in ripening reversion in *Cnr* fruits (Chen *et al.*, 2015*b*). Moreover, VIGS of *SlSPL-CNR* leads wild-type tomato (*Solanum lycopersicum* cv. Ailsa Craig, AC) to phenocopy the physical, physiological, biochemical, and molecular characteristics of *Cnr* fruits (Lai *et al.*, 2015).

The *SPL* gene family consists of a group of genes encoding the SBP-box TFs, which are unique to plants (Cardon *et al.*, 1999; Zhang *et al.*, 2015). SBP-box genes were previously identified in *Antirrhinum majus* and their protein products bind to the promoter of the floral meristem identity gene *SQUAMOSA* (Huijser *et al.*, 1992; Klein *et al.*, 1996). Subsequently many SBP-box genes have been identified in at least 66 organisms from green algae to flowering plants (Cardon *et al.*, 1999; Zhang *et al.*, 2015). In tomato, 15 members of the SBP-box gene family have been reported, although most of them are not functionally characterized. Of the SBP-box genes identified to date, *SlSPL-CNR* is closely related to the tomato *SlySBP3* (*SlSBP3*), potato *StSBP3*, and Arabidopsis *AtSPL3* genes (Salinas *et al.*, 2012). In plants, SBP-box genes are involved in different growth and development processes such as microsporogenesis and megasporogenesis (Unte *et al.*, 2003), kernel development (Wang *et al.*, 2005), male inflorescence size (Wu *et al.*, 2016*a*), male fertility (Xing *et al.*, 2010, 2013), plant architecture (Stone *et al.*, 2005), floral transition (Cardon *et al.*, 1997), lateral primordia initiation (Chuck *et al.*, 2014), leaf development (Yamaguchi *et al.*, 2009; Hou *et al.*, 2017), bract development and meristem boundaries (Chuck *et al.*, 2010; Preston and Hileman, 2010), shoot maturation (Schwarz *et al.*, 2008; Shikata *et al.*, 2009), ovary and fruit development (Manning *et al.*, 2006; Ferreira e Silva *et al.*, 2014; Chen *et al.*, 2015*a*), as well as ear development and yields (Zhang *et al.*, 2014; Wu *et al.*, 2016*b*; Wang and Zhang, 2017; Zhang *et al.*, 2017). SBP-box TFs are diverse in their primary protein structures but share a highly conserved DNA-binding domain of approximate 80 amino acid (aa) residues. Moreover, the Arabidopsis SlSPL-CNR orthologues AtSPL4 and AtSPL7 possess a zinc-finger motif (ZFM; Yamasaki *et al.*, 2004) and within the SBP domain there is a bipartite nuclear localization signal (NLS; Birkenbihl *et al.*, 2005). It is also established that the SPL-family TFs such as *A. majus* AmSBP1 and AmSBP2 (Klein *et al.*, 1996), AtSPL3 (Cardon *et al.*, 1997), AtSPL4, AtSPL7 (Yamasaki *et al.*, 2004), and AtSPL8 (Birkenbihl *et al.*, 2005), and the single-cell alga *Chlamydomonas* CRR1 (Birkenbihl *et al.*, 2005) bind *in vitro* to the *A. majus SQUAMOSA* and the orthologous Arabidopsis *AP1* promoters.

On the other hand, *SnRK* represents a family of genes encoding SNF1-RELATED PROTEIN KINASES that act as a global regulator of carbon metabolism. In plants the *SnRK* family has been grouped into three subfamilies, namely *SnRK1*, *SnRK2*, and *SnRK3* (Coello *et al.*, 2011). Similar to SBP-box TF genes, *SnRKs* play essential roles in various physiological processes such as leaf senescence (Kim *et al.*, 2017), early kernel development (Bledsoe *et al.*, 2017), pollen hydration (Gao *et al.*, 2016; Liu *et al.*, 2017) and development (Zhang *et al.*, 2001), cellular energy homeostasis and cell proliferation (Guérinier *et al.*, 2013), biotic and abiotic stress (Cho *et al.*, 2012; Lin *et al.*, 2014; Perochon *et al.*, 2015), cell death and hypersensitive response (Szczesny *et al.*, 2010; Avila *et al.*, 2012), herbivory tolerance (Schwachtje *et al.*, 2006), seed germination and seedling growth (Lu *et al.*, 2007), and crop yield (Lawlor and Paul, 2014). *SnRK1* has been found to be involved in anthocyanin accumulation in apple (Liu *et al.*, 2017) and tomato (Wang *et al.*, 2012) fruit development. More recently, it has been reported that *SnRK2* negatively influences fruit development and ripening in strawberry (Han *et al.*, 2015).

In this article, we report on the molecular and functional dissection of SlSPL-CNR. Using PCR-based site-directed mutagenesis and a potato virus X (PVX)-based transient gene expression system, we reveal that SlSPL-CNR is localized to the nucleus through a distinct monopartite NLS and binds to zinc. The NLS is required for SlSPL-CNR to trigger plant cell death, but ZFMs may contribute. SlSPL-CNR requires both NLS and ZFMs to complement ripening in *Cnr* fruits. Using yeast-two-hybrid screening and a co-immunoprecipitation (CoIP) assay, we identified SlSnRK1 as a SlSPL-CNR-interacting protein. VIGS of *SlSnRK1* affects expression of a spectrum of ripening-related genes and inhibits ripening in tomato. These results shed light on how SlSPL-CNR acts in tomato fruit ripening. Moreover, our findings also demonstrate that SlSPL-CNR is a multi-functional protein capable of triggering cell death in plants.

## Materials and methods

### Plant materials and growth

Wild-type tomato (*S. lycopersicum* cv. Ailsa Craig (AC)) and *Nicotiana benthamiana* plants were grown in insect-free growth rooms or greenhouses at 25 °C under a 16 h light–8 h dark cycle with a humidity of 60–80%.

### Construct

Virus transient vectors to express mutant SlSPL-CNR:GFP fusion proteins were generated as previously described (van Wezel *et al.*, 2002). Briefly, mutant SlSPL-CNR coding sequences (Supplementary Fig. S1; Supplementary Table S1) were amplified by either standard PCR or overlapping PCR using primers listed in Supplementary Table S2, and cloned into the PVX/green fluorescent protein (GFP) vector to produce PVX/SlSPL-CNR mutant:GFPs (Supplementary Figs S2, S3; Supplementary Table S2). PVX/SlSPL-CNR:GFP was generated previously (Manning *et al.*, 2006). To express free SlSPL-CNR protein, the wild-type *SlSPL-CNR* gene was amplified with PP298 (5′-CCTCAC*AtcGAT*GGAAACTAACAAATGGGAAGGGA-3′, *Cla*I italicized) and the 3′-end primer (5′-GATGCT*cggcCg*TCAGCCCAAATTTTCTCCATGAGAG-3′, *Eag*I italicized), and cloned into the *Cla*I/*Eag*I sites of the PVX vector (van Wezel *et al.*, 2002) to generate PVX/SlSPL-CNR. A 500 bp fragment of the *SlSnRK1* gene was amplified by PCR using a cDNA library prepared from the tomato fruit pericarp and cloned to the PVX vector to produce PVX/SlSnRK1 (Supplementary Dataset S1). All constructs were verified by DNA sequencing.

### Virus transient gene expression and virus-induced gene complementation

Virus transient gene expression was carried out in repeated experiments as previously described (Qin *et al.*, 2017). In each experiment, three to six young AC or *N. benthamiana* plants were mock-inoculated or inoculated with recombinant PVX RNAs produced by *in vitro* transcription. Virus-induced gene complementation (VIGC) in *Cnr* fruits was performed as previously described (Zhou *et al.*, 2012).

### Epifluorescence and confocal microscopy

Virus-inoculated AC or *N. benthamiana* was routinely examined under long-wave length ultraviolet light (Upland UVP Model B 100AP) to check transient GFP expression and systemic spread of the recombinant viruses. Photographs were taken with a Zeiss Axiophot microscope with filters (excitation at 450–490 nm and long-pass emission at 520 nm or excitation at 546 nm and long-pass emission at 590 nm) through a Nikon Coolpix 995 digital camera (Li *et al.*, 2011). Confocal imaging of the leaves was performed with a Zeiss LSM 710 three-channel microscope with an excitation light of 405 nm, and the emission was captured at 454–581 nm.

### Zinc-afﬁnity pull-down and western blot

Young leaf tissues were collected at 14 d post-inoculation, ground in liquid nitrogen and resuspended in extraction buffer (50 mM Tris–HCl (pH 8.0), 1 mM phenylmethylsulfonyl ﬂuoride) containing 0, 100, or 400 mM NaCl. Insoluble debris was discarded after centrifugation, and supernatants were collected. Zinc-afﬁnity pull-down assays were performed as described (van Wezel *et al.*, 2003). Briefly, an equal amount of wild-type or SlSPL-CNR mutant:GFP fusion protein in 0, 100, or 400 mM NaCl was incubated with a 50 μl aliquot of zinc chelate afﬁnity resin (iminodiacetic acid Sepharose 6B; Sigma-Aldrich) pre-equilibrated with the extraction buffer containing 0, 100, or 400 mM NaCl, as appropriate. Resins were then washed three times with the same buffer, resuspended in 100 μl gel loading buffer, and boiled for 3 min before loading samples onto a sodium dodecyl sulphate–15% polyacrylamide gel. After electrophoresis, proteins were immobilized on nitrocellulose membranes and immune-detected by use of a SlSPL-CNR or GFP antibody (van Wezel and Hong, 2004).

### Yeast two-hyrbid screening

Matchmaker Gold Yeast Two-Hybrid System (PT4084-1, Clontech, USA) was performed following the manufacturer’s guidance with minor modifications. Briefly, the SlSPL-CNR coding region was PCR amplified using a pair of primers (Y2H_SlSPL-CNR-F: 5′-GAGTCGGAATTCATGGAAACTAACAAATGGGAAGGG-3′ and Y2H_SlSPL-CNR-R: 5′-TCGACAGGATCCTCAGCCCAAATTTTCTCCATGAGAG-3′), and cloned into the *Eco*RI/*Bam*HI sites of the pGBKT7 vector to generate the bait construct pGBKT7/SlSPL-CNR (Supplementary Figs S4, S5). The integrity of this construct was confirmed by sequencing. For construction of a tomato cDNA library, total RNA was extracted from the pericarp tissues of AC fruits at the breaker stage using an RNAeasy Plant Mini Kit (Qiagen, Germany). Then, oligo dT-primed cDNAs were generated using the Make Your Own ‘Mate & Plate’ Library System (PT4085, Clontech, USA-1). Amplification of SMART (Switching Mechanism at 5′ end of RNA Transcript) cDNAs by long distance PCR was performed using the Advantage 2 Polymerase Mix, and one set of products was size-selected using CHROMA SPIN+TE-400 columns following the protocol of Clontech’s SMART technology. Finally, a sequence homologous to the prey vector pGADT7-Rec was added to a pool of double-stranded (ds) cDNAs. The purified SMART dscDNA, pGADT7-Rec AD Cloning Vector (*Sma*I-linearized) and pGBKT7/SlSPL-CNR were co-transformed into yeast strain AH109 using the Yeastmaker Yeast Transformation System 2 (PT1172-1, Clontech, USA). An aliquot of suspensions of the transformation mixture was spread evenly onto 150 mm plates with SD/−Trp, SD/−Leu/−Trp or SD/−Ade/−His/−Leu/−Trp medium. After incubation at 30 °C for 3–5 d, positive colonies were identified and prey plasmids were extracted by a TIANprep Yeast Plasmid DNA Kit (Tiangen, China). Inserted cDNA in the pGADT7-Rec vectors was identified by PCR amplification using the T7-primer (5′-TAATACGACTCACTATAGGGC-3′) and the AD-primer (AGATGGTGCACGATGCACAG), then sequenced and analysed using an online blast programme (http://blast.ncbi.nlm.nih.gov). A yeast β-galactosidase assay was performed following the manufacture’s *Yeast Protocols Handbook* (Clontech Laboratories, Inc.). Student’s *t*-test was carried out against the negative controls (http://www.physics.csbsju.edu/stats/t-test.html).

To investigate whether the intact SlSnRK1 protein would interact with SlSPL-CNR in yeast, the full-length coding sequence for SlSnRK1 was amplified using the tomato cDNA library as template and a specific set of primers, and cloned into the pGBKT7 and pGADT7 vectors (Supplementary Fig. S6; Supplementary Table S4). An extra pGADT7/SlSPL-CNR was also constructed (Supplementary Fig. S6; Supplementary Table S4). A yeast two-hybrid (Y2H) assay for testing SlSnRK1/SlSPL-CNR interaction was performed as described above.

### Agroinflitration and co-immunoprecipitation assay

We constructed pCAMBIA1300/35S-eGFP, pCAMBIA1300/35S-FLAG, pCAMBIA1300/35S-SlSPL-CNR:eGFP and pCAMBIA1300/35S-SlSnRK1:FLAG in the binary pCAMBIA1300 vector (Yu *et al.*, 2018) in order to express free GFP, 3×FLAG, GFP-tagged SlSPL-CNR and 3×FLAG-tagged SlSnRK1 proteins in plants (Supplementary Fig. S7A; Supplementary Table S4). These binary gene expression constructs were respectively transformed into *Agrobacterium tumefaciens* GV3101. Two young leaves per *N. benthamiana* plant at the six-leaf stage were infiltrated or co-inﬁltrated with 1 OD_600_ agrobacteria harbouring different gene expression vectors in repeated experiments as described (Chen *et al.*, 2018*b*). Agro-infiltrated leaf tissues were collected at 3 d post-infiltration for further analysis. For analysis of protein expression, total proteins were extracted from *N. benthamiana* leaves (1 g leaf tissues for each sample) using the Plant Protein Extraction Kit (CWBIO, www.cwbiotech.com). Protein gel separation and western blot were performed as described above using either anti-GFP (Abcam) or anti-FLAG antibody (Sigma-Aldrich). A CoIP assay was performed using ANTI-FLAG^®^ M2 Magnetic Beads (Sigma-Aldrich). Briefly, total proteins were extracted from *N. benthamiana* leaves (1 g leaf tissues for each sample) in ice-cold buffer (50 mM Tris–HCl, pH 7.4, with 150 mM NaCl, 1 mM EDTA, and 1% Triton X-100). Protein extracts were then incubated with ANTI-FLAG^®^ M2 Magnetic Beads for 12 h at 4 °C. The precipitations were washed four times with ice-cold immunoprecipitation buffer (50 mM Tris–HCl, 150 mM NaCl, pH 7.4) at 4 °C and were analysed by immunoblot using anti-GFP antibody (Abcam).

### Virus-induced gene silencing

PVX-based VIGS of *SlSnRK1* expression was performed in AC fruits at various developmental stages on different trusses on the same plants, and on different plants in repeated experiments as described (Manning *et al.*, 2006). In each experiment, pedicels of 30–40 fruits at 5–20 d post-anthesis (DPA) were mock-injected with Tris–EDTA buffer or injected with PVX/SlSnRK1 transcripts. Tomato plants were grown and maintained in growth rooms at 25 °C with supplementary lighting to give a 16 h photoperiod. Fruits were daily examined and photographed with a Nikon Coolpix 995 digital camera.

### RT-PCR and qRT-PCR

Total RNA was extracted from *N. benthamiana* leaf tissues or AC pericarp tissues using the RNAeasy Plant Mini Kit (Qiagen). First-strand cDNA was synthesized using equal amounts of total RNA and a FastQuant RT Kit with gDNA Eraser (Tiangen). RT-PCR was performed as previously described (Li *et al.*, 2011). Real-time PCR was performed using a CFX96 Real-Time system (Bio-Rad) with the UltraSYBR Mixture (CoWin Bioscience) and gene-specific primers (Supplementary Table S2; Supplementary Dataset S1). 18S rRNA was used as an internal control, and at least three biological duplicates and four technical duplicates per biological duplicate were used for each of repeated experiments. The relative expression level was calculated by the 2^−∆∆*C*t^ method as described (Livak and Schmittgen, 2001; Qin *et al.*, 2012). To analyse gene expression in VIGSed fruits, we dissected the green non-ripe and red ripening sectors and extract total RNAs from each sector. These RNAs were used in qRT-PCR assays along with three different sets of primers (Supplementary Dataset S1) in order to examine how VIGS affected the level of *SlSnRK1* mRNA transcripts. The relative expression level in the green or red sectors of VIGSed fruits was further normalized against the level of *SlSnRK1* mRNA in AC fruits at Breaker+5 d (B+5). RT-qPCR data between ripe and non-ripe sectors were analysed by Student’s *t-*test (http://www.physics.csbsju.edu/stats/t-test.html). The statistical signiﬁcance threshold was *P*≤0.05.

### DNA methylation assay

Whole genome bisulfite sequencing data were previously generated in our laboratory (Chen *et al.*, 2015*b*) or available online (Zhong *et al.*, 2013). Characterization of DNA methylation profiles was performed as previously described (Chen *et al.*, 2015*b*).

## Results

### SlSPL-CNR is a nucleus-localized protein and can trigger cell death in tomato

**Fig. 1.**
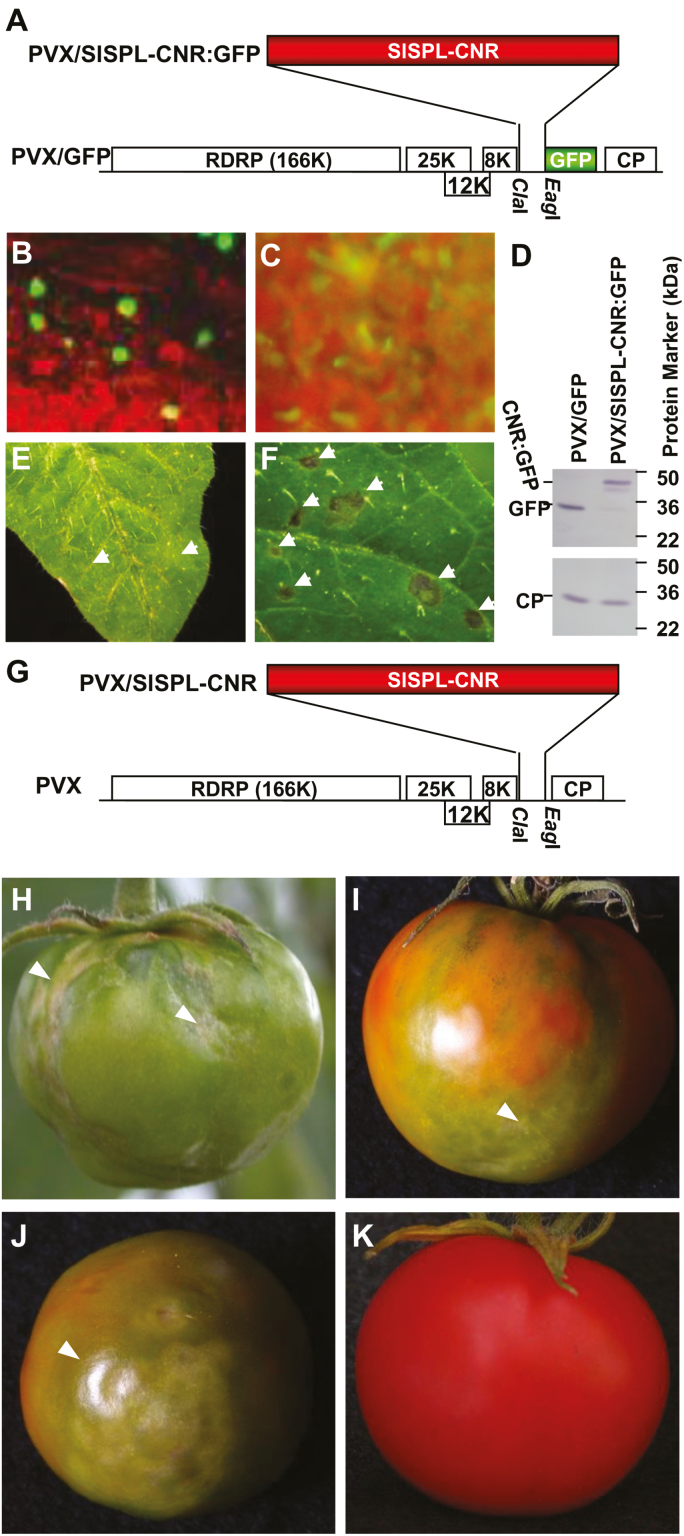
Expression of SlSPL-CNR induces necrotic cell death. (A) Diagrammatic representation of viral transient gene expression vector PVX/SlSPL-CNR:GFP. Genome organization of PVX/GFP and two cloning sites are indicated. The 166K RDRP is the viral RNA-dependent RNA polymerase. The triple-gene block encodes three viral movement proteins of 25, 12, and 8 kDa. GFP was fused in-frame to SlSPL-CNR to express a fusion protein. CP is the viral coat protein. (B) Nuclear localization of SlSPL-CNR:GFP in tomato leaf epidermal cells. (C) Cytoplasmic localization of free GFP protein in tomato leaf epidermal cells. Photographs were taken under an epifluorescence microscope at 7 d post-inoculation (dpi). (D) Western blot detection of SlSPL-CNR:GFP fusion protein. Protein samples were extracted from young tomato leaf tissues at 14 dpi. Immuno-detection was performed using either a GFP antibody (upper panel) or a PVX CP antibody (lower panel). (E, F) Induction of necrotic cell death in tomato leaf tissues. Tomato leaves inoculated with PVX/GFP (E) or PVX/SlSPL-CNR (F) developed chlorotic or necrotic lesions, respectively. Photographs were taken at 7 dpi. (G–K) Induction of necrotic cell death in tomato AC fruits. AC fruits injected with PVX/SlSPL-CNR (G) developed necrosis at different stages including mature green (H), breaker/colour turning (I) and ripening (J). An AC fruit infected with PVX/GFP (A) ripened and remained healthy (K). All fruits were photographed at 33 d post-injection.

We used PVX/SlSPL-CNR:GFP (Fig. 1) to express the SlSPL-CNR (15 kDa) and GFP (27 kDa) fusion protein in *S. lycopersicum* AC plants, and found that green fluorescence was predominantly confined within the nuclei of tomato leaf cells (Fig. 1A, B). In contrast, we observed fluorescence of free GFP throughout the cytoplasm in cells of tomato leaf tissues infected with PVX/GFP (Fig. 1C). Viral expression of SlSPL-CNR:GFP fusion protein (42 kDa) and free GFP was detected. PVX infection of AC leaf tissues was further evidenced by immunodetection of viral coat protein (CP; Fig. 1D). These data demonstrate that the PVX-based transient gene expression system was effective to express SlSPL-CNR:GFP in tomato cells, and that SlSPL-CNR is a nucleus-localized protein. We also observed that PVX/GFP induced chlorotic lesions, typical local symptoms associated with PVX infection (Fig. 1E), whilst virally expressed SlSPL-CNR:GFP elicited cell death and produced severe necrotic lesions on the inoculated AC leaves (Fig. 1F). However, we did not observe cell death in AC fruits that were injected with PVX/SlSPL-CNR:GFP (Manning *et al.*, 2006), likely due to fusion of GFP having a negative influence on SlSPL-CNR activity. However, AC fruits treated with PVX/SlSPL-CNR (Fig. 1G) that are expected to express free SlSPL-CNR protein with full functionality developed necrotic cell death (Fig. 1H–J), whilst control AC fruits treated with PVX/GFP remained normal (Fig. 1K).

### SlSPL-CNR comprises a distinct monopartite _30_KRKR_33_ NLS

**Fig. 2.**
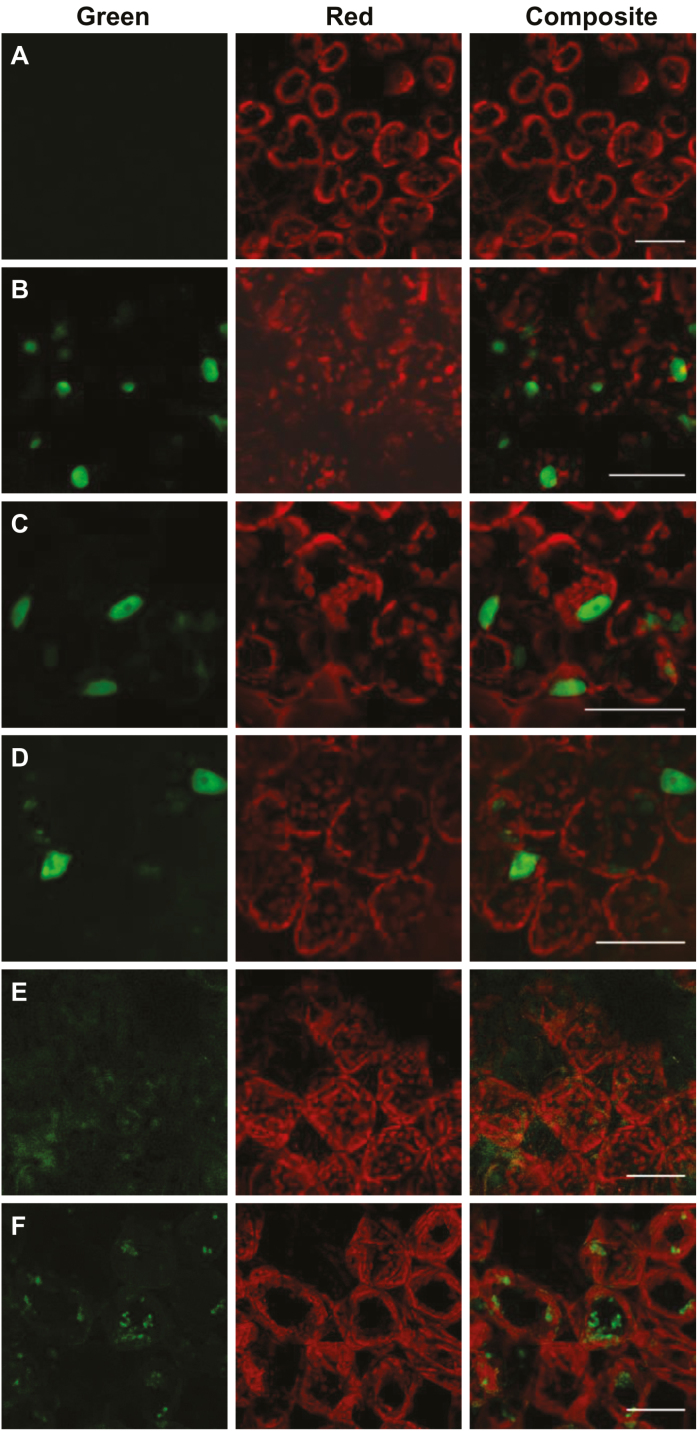
Characterization of the nuclear localization signal for SlSPL-CNR. (A) Mock*-*inoculated *N. benthamiana* (*Nb*) leaf cells as a negative control. (B–F) *Nb* leaf cells expressing SlSPL-CNR:GFP (B), SlSPL-CNR123:GFP (C), SlSPL-CNR1235:GFP (D), SlSPL-CNR4:GFP (E), or SlSPL-CNR12345:GFP (F). *Nb* leaves were taken at 7 d post-inoculation and examined under a confocal microscope. Scale bar: 100 μm.

SlSPL-CNR consists of 136 aa residues. Similar to other SPB-box TFs, SlSPL-CNR consists of a lysine/arginine (K/R)-rich region, _109_*KR*SC*RRR*LAGHNE*RRRK*_125_, at its C-terminus. We designated residues _109_KR_110_, _113_RRR_115_, and _122_RRRK_125_ as domain I, II, and III, respectively (Supplementary Fig. S1). Domain I and domain III within this region represent a bipartite NLS for several SBP-box TFs (Birkenbihl *et al.*, 2005). To test whether SlSPL-CNR has a similar bipartite NLS, we mutated _109_KR_110_ and _122_RRRK_125_ by replacing the six K/R residues with alanine (A) for virally expressing SlSPL-CNR13:GFP in *N. benthamiana* (Supplementary Fig. S2). Compared with the negative control (mock inoculation; Fig. 2A), SlSPL-CNR13:GFP was found to localize in the cell nucleus, similar to wild-type SlSPL-CNR:GFP fusion protein (Fig. 2B; Supplementary Table S1).

We then produced PVX constructs to express SlSPL-CNR1:GFP, SlSPL-CNR2:GFP, SlSPL-CNR3:GFP, SlSPL-CNR12:GFP, SlSPL-CNR23:GFP, and SlSPL-CNR123:GFP mutant proteins, of which each of the individual domains (I, II, or III) or combinations was replaced with alanine (Supplementary Fig. S2). Similar to SlSPL-CNR:GFP (Fig. 2B), the single- or double-domain mutated proteins were found to be all cell nucleus-localized (Supplementary Table S1). The triple-domain mutant protein SlSPL-CNR123:GFP was also predominantly restricted to the cell nucleus (Fig. 2C; Supplementary Table S1). These data indicate that the bipartite NLS shown previously for several SBP-box TFs (Birkenbihl *et al.*, 2005) and the three-consecutive arginine (_103_RRR_105_) residues do not contribute to a functional NLS that determines the nuclear localization of SlSPL-CNR.

This unexpected finding stimulated further examinations of the SlSPL-CNR protein sequence, which revealed two extra basic amino acid-rich domains, _30_KRKR_33_ and _68_HRRHK_72_ (dubbed IV and V, respectively; Supplementary Fig. S1). We then constructed an extra 25 viral vectors to express SlSPL-CNR:GFP fusion proteins in which the five basic amino acid domains were mutated in every possible permutation in order to identify a functional NLS for SlSPL-CNR (Supplementary Fig. S2). Outcomes of these experiments are summarized in Supplementary Table S1 and representatives of confocal microscopic images are shown in Fig. 2.

We found that SlSPL-CNR mutants, which maintained domain IV, retained the functionality to translocate the GFP fusion protein to the cell nucleus (Supplementary Table S1). For instance, as SlSPL-CNR123:GFP (Fig. 2C), green fluorescence of SlSPL-CNR1235:GFP, in which all K/R residues in domains I, II, III, and V were replaced with A, was predominantly present in the cell nucleus (Fig. 2D). On the other hand, SlSPL-CNR derivatives, as long as their domain IV was mutated, were no longer nuclear-localized (Supplementary Table S1). Indeed, the single domain IV-mutated SlSPL-CNR4:GFP failed to localize to the nucleus and its GFP fluorescence was present in the cytoplasm (Fig. 2E). A similar cytoplasmic appearance of GFP fluorescence was observed for SlSPL-CNR12345:GFP, a quint-mutant protein in which the five basic amino acid-rich domains were all mutated (Fig. 2F). Taken together, these results demonstrate that _30_KRKR_33_ (domain IV) at the N-terminus represents a distinct monopartite NLS that determines the nuclear localization of SlSPL-CNR in plant cells.

### Requirement of NLS for SlSPL-CNR to induce cell death

**Fig. 3.**
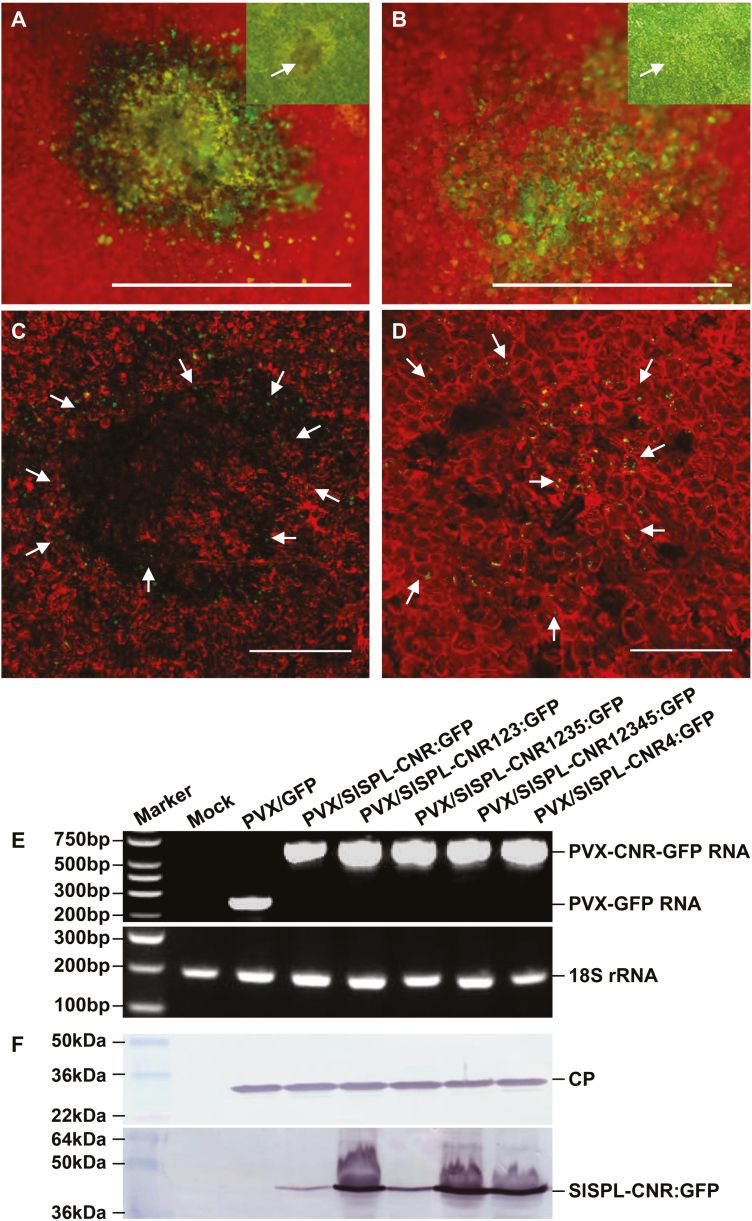
Requirement of a functional NLS for SlSPL-CNR to induce necrotic cell deaths. (A–D) Representative images of necrotic and chlorotic lesions. Necrotic cell death is associated with the wild-type SlSPL-CNR:GFP protein (A, C). Chlorotic lesions consist of healthy cells expressing the SlSPL-CNR4:GFP protein (B, D). Photographs of lesions/leaf cells were taken at 7-d post-inoculation (dpi) under an epifluorescence microscope (A, B) or confocal microscope (C, D). The inset images of a necrotic cell death lesion in (A) and a chlorotic lesion in (B) were photographed under normal light. GFP fluorescence is green and chlorophyll autofluorescence is red. Necrotic dead tissues appear yellow. Scale bar: 1 mm (A, B), 500 nm (C, D). Arrows indicate either nuclear or cytoplasmic localization of SlSPL-CNR:GFP (C) or SlSPL-CNR4:GFP (D). (E) RT-PCR detection of recombinant PVX RNA or 18S rRNA as indicated. RNA samples were extracted from young leaf tissues at 14 dpi. Sizes and positions of DNA ladders as well as positions of target genes are indicated. (F) Western blot detection of PVX CP and the wild-type and mutant SlSPL-CNR:GFP fusion proteins. Upper panel, CP antibody; lower panel, SlSPL-CNR antibody. Sizes and positions of protein markers as well as CP and SlSPL-CNR:GFP fusion protein are indicated.

Expression of the wild-type SlSPL-CNR protein triggered severe necrosis and cell death in tomato leaf tissues. We then investigated how plants responded to the NLS-mutated SlSPL-CNRs (Fig. 3). SlSPL-CNR:GFP or 31 SlSPL-CNR mutant–GFP fusion proteins (Supplementary Fig. S2) were respectively expressed and a typical necrotic or chlorotic lesion is shown (Fig. 3A–D). Extensive necrosis was found in the lesions resulting from PVX/SlSPL-CNR:GFP infection with many broken chloroplasts observed in dying and dead cells (Fig. 3A, C). Nevertheless, GFP fluorescence of SlSPL-CNR:GFP was observed predominantly in nuclei of cells around the periphery of the necrotic lesion (Fig. 3A, C). In contrast, healthy cells with intact chloroplasts were found in the lamina of the chlorotic lesion associated with PVX/SlSPL-CNR4:GFP infection. Consistent with this, SlSPL-CNR4:GFP was no longer nucleus-localized and the GFP fluorescence was observed in the cytoplasm (Fig. 3B, D). SlSPL-CNR mutant proteins that had lost the functional NLS (_30_KRKR_33_) lost the capability to induce cell death, whilst these nucleus-localized mutant proteins maintained their activity to trigger cell death (Supplementary Table S1).

This finding was supported by the analysis of accumulation of the recombinant PVX RNAs (Fig. 3E), viral CP and SlSPL-CNR:GFP fusion proteins (Fig. 3F). No viral RNA, CP or SlSPL-CNR protein was detected in mock-inoculated plants. However, specific recombinant *PVX-GFP* or *PVX-CNR-GFP* RNAs were detected in virus-infected leaf tissues (Fig. 3E). Consistently, viral CP was detected in all virus-infected plants. However, wild-type or mutant SlSPL-CNR:GFP fusion proteins were not detected in mock-inoculated or PVX/GFP-infected plants, but were readily detectable in plants in which SlSPL-CNR:GFP, SlSPL-CNR123:GFP, SlSPL-CNR1235:GFP, SlSPL-CNR12345:GFP, or SlSPL-CNR4:GFP was expressed (Fig. 3F).

### SlSPL-CNR binds to zinc and the zinc-binding activity contributes to SlSPL-CNR-mediated induction of cell death

**Fig. 4.**
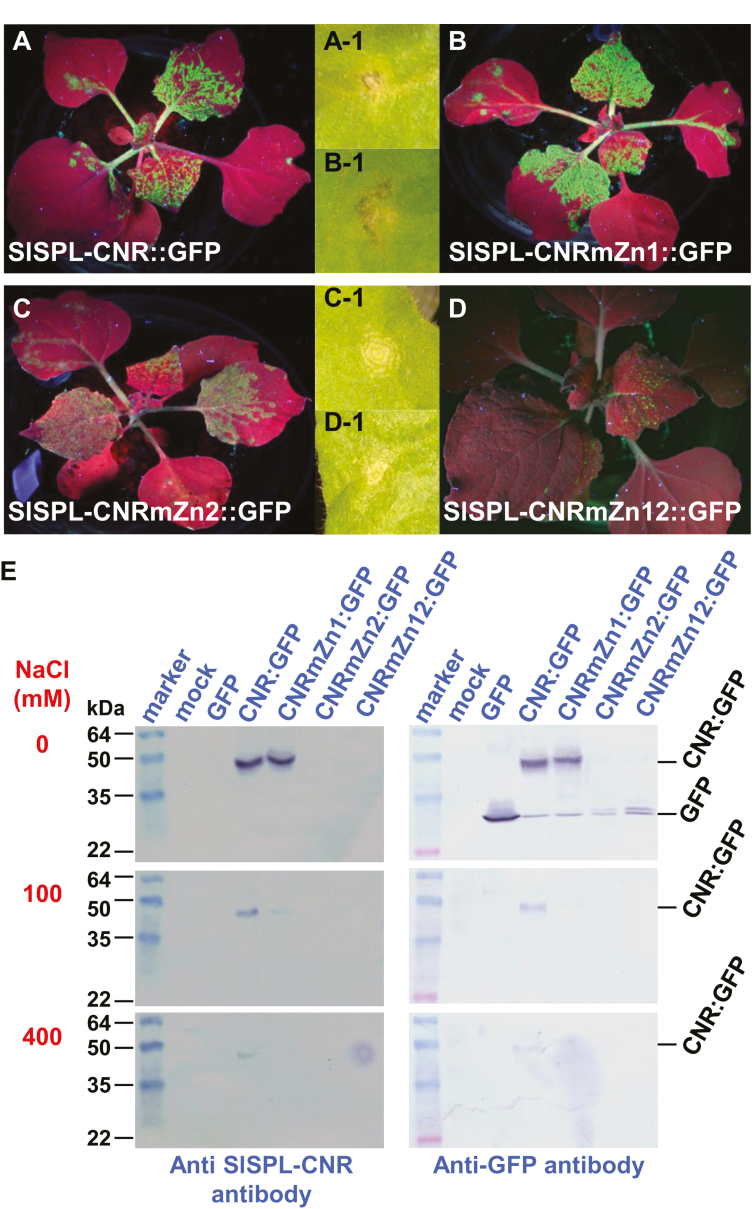
Involvement of zinc-finger motif in induction of necrotic cell death. (A–D) Impact of mutations in zinc-finger motifs on SlSPL-CNR in triggering severe necrosis. Expression of SlSPL-CNR:GFP (A), SlSPL-CNRmZn1:GFP (B), SlSPL-CNRmZn2:GFP (C), or SlSPL-CNRmZn12:GFP (D) is indicated by the GFP fluorescence in young leaves. Severe necrosis (A-1, B-1) and mild necrotic ringspot (C-1, D-1) are indicated for each of the corresponding fusion proteins. Entire plants were photographed under long-wavelength UV light at 14 d post-inoculation (dpi), whilst lesions were photographed under white light at 7 dpi. (E) Zinc-affinity pull-down assay. Proteins were detected using either anti-SlSPL-CNR or GFP antibody as indicated. The SeeBlue™ Plus2 Pre-stained Protein Standard (Thermo Fisher Scientific) was included in gels. Sizes and positions of protein markers are indicated. SlSPL-CNR:GFP fusion (CNR:GFP, 42k Da) and GFP free protein (27 kDa) as well as NaCl concentration (mM) used in the washing buffer are also indicated.

The SlSPL-CNR protein is predicted to possess two putative ZFMs, named Zn1 and Zn2, within the conserved SBP domain (Supplementary Fig. S1). To test whether Zn1 and Zn2 are required for SlSPL-CNR to bind to zinc, we expressed SlSPL-CNR:GFP (wild-type), Zn1- or Zn2-mutated protein SlSPL-CNRmZn1:GFP or SlSPL-CNRmZn2:GFP, or Zn1/Zn2 double-mutant protein SlSPL-CNRmZn12:GFP (Fig. 4; Supplementary Fig. S3; Supplementary Table S2). Viral expression of these proteins was evident by the occurrence of the GFP fluorescence in *N. benthamiana* (Fig. 4A–D). SlSPL-CNRmZn1:GFP acted like SlSPL-CNR:GFP to induce severe necrotic cell death (Fig. 4A-1, B-1). However both SlSPL-CNRmZn1:GFP and SlSPL-CNRmZn12:GFP were only able to produce mild necrotic ringspots (Fig. 4C-1, D-1).

Through zinc-afﬁnity pull-down assays, we found that SlSPL-CNR:GFP and the Zn1 mutant protein bound sufficiently to zinc under no-NaCl conditions. The wild-type protein remained bound to zinc at 100 or 400 mM NaCl. However, the zinc-binding ability of SlSPL-CNRZn1:GFP was reduced at 100 mM NaCl, and no binding was found in the high salt (400 mM NaCl; Fig. 4E, left and right panels). Strikingly, both the Zn2 and Zn1/Zn2 single or double mutants almost completely lost their zinc-binding ability. Only a trace amount of Zn2 and Zn1/Zn2 mutant proteins was detected in the no-salt buffer (Fig. 4E, right panel). We also observed slight degradation of SlSPL-CNR:GFP, SlSPL-CNRmZn1:GFP, SlSPL-CNRmZn2:GFP, or SlSPL-CNRmZn12:GFP, evidenced by detection of a band of the similar size of free GFP (Fig. 4E, top right panel). Taken together, our findings demonstrate that SlSPL-CNR is a zinc-binding protein and the Zn2 motif makes a limited contribution to the induction of plant cell death.

### Requirement of functional NLS and ZFMs for SlSPL-CNR to complement *Cnr* mutant

**Fig. 5.**
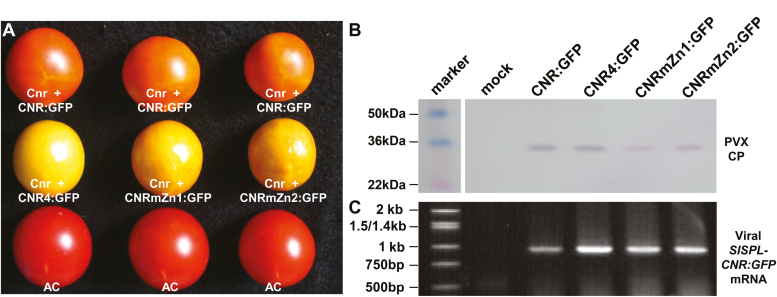
Requirement of functional nuclear localization signal and zinc finger motifs in SlSPL-CNR-mediated ripening reversion in *Cnr* fruits. (A) Virus-induced gene complementation in the *Cnr* fruits. Representative *Cnr* fruits that were injected with PVX/SlSPL-CNR:GFP (Cnr+CNR:GFP) were ripe. These *Cnr* fruits that were injected with PVX/SlSPL-CNR4:GFP (Cnr+CNR4:GFP), PVX/SlSPL-CNRmZn1:GFP (Cnr+CNRmZn1:GFP), or PVX/SlSPL-CNRmZn2:GFP (Cnr+CNRmZn2:GFP) remained colourless non-ripening. Wild-type AC fruits were included as positive controls. Fruits were photographed at 45 d post-anthesis. (B, C) Western blot detection of PVX CP and RT-PCR assays of viral transient SlSPL-CNR:GFP mRNA in *Cnr* fruits. Fruits were mock-treated or injected with recombinant PVXs as indicated in (A). Sizes and positions of protein markers and the 1 kb DNA ladder as well as PVX CP and viral SlSPL-CNR:GFP mRNA are indicated.

To assess whether SlSPL-CNR requires the monopartite NLS and the two ZFMs to influence fruit ripening, we exploited a VIGC assay (Zhou *et al.*, 2012) to express wild-type, NLS- or ZFM-mutated SlSPL-CNR in *Cnr* fruits (Fig. 5). Similar to our previous analysis (Kong *et al.*, 2013), approximately 15% of *Cnr* fruits that were injected with PVX/SlSPL-CNR:GFP turned orange-red (Fig. 5A), suggesting that the wild-type SlSPL-CNR expressed from the recombinant virus could at least partially complement and lead the *Cnr* mutant fruits to ripen to a certain degree. However all *Cnr* fruits that were injected with PVX/SlSPL-CNR4:GFP, PVX/SlSPL-CNRmZn1:GFP, or PVX/SlSPL-CNRZn2 remained non-ripe, showing the typical ‘colourless non-ripening’ phenotypes (Fig. 5A). The presence of the respective recombinant viruses and expression of the wild-type or mutant SlSPL-CNR mRNA in the *Cnr* fruits were readily detected either by western blot using the PVX CP antibody or by RT-PCR (Fig. 5B, C). These findings demonstrate that functional NLS and ZFMs are required for SlSPL-CNR to carry out its proper activity to induce ripening reversion in the *Cnr* fruits.

### SlSnRK1 interacts with SlSPL-CNR

**Fig. 6.**
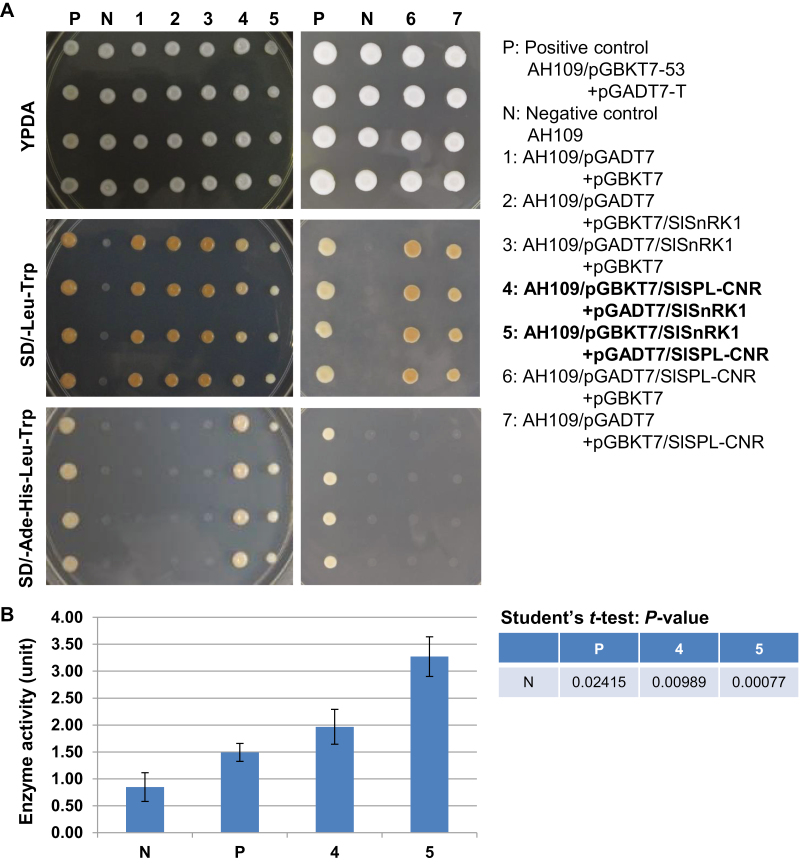
Interactions between SlSPL-CNR and SlSnRK1. (A) Interactions between SlSPL-CNR and SlSnRK1 in two Y2H conformations. P, positive control—yeast strain AH109 carrying both pGBKT7-53 and pGADT7-T. N, negative control—AH109 strain only. Samples 1–7 are indicated. Yeast was cultured on YPDA agar plates (YPDA), synthetically defined (SD) medium plate without supplement of leucine (Leu) and tryptophan (Trp; SD/−Leu−Trp), or SD without supplement of adenine (Ade), histidine (His), Leu, and Trp (SD/−Ade−His−Leu−Trp). Positive interaction between SlSPL-CNR and SlSnRK1 resulted in AH109 growth in SD/−Ade−His−Leu−Trp plates (P; samples 4 and 5). (B) Quantitative analysis of protein–protein interactions using β-galactosidase activity assay. β-Galactosidase assays were performed following Clontech’s protocol. One unit of β-galactosidase is defined as the amount that hydrolyses 1 µmol of *o*-nitrophenyl β-D-galactopyranoside to *o*-nitrophenol and D-galactose per min per cell. Samples are indicated as in (A). Three biological duplicates (*n*=3) for each sample in two separate experiments were used in the β-galactosidase assays (mean ±SD). Student’s *t*-test was carried out against the negative control (N). *P*-values are indicated. The statistically significant increases in the β-galactosidase activity in AH109 co-transformed with pGBKT7/SlSPL-CNR+pGADT7/SlSnRK1 or pGBKT7/SlSnRK1+pGADT7/SlSPL-CNR confirm positive interactions between SlSPL-CNR and SlSnRK1.

To understand how SlSPL-CNR affects fruit ripening in tomato, we used SlSPL-CNR as bait (Supplementary Fig. S4A) to screen a tomato fruit prey cDNA library (Supplementary Fig. S4B) in a Y2H system to identify SlSPL-CNR-interacting proteins. We obtained 80 positive yeast colonies for DNA sequencing (Supplementary Fig. S4C–E) and produced 47 good sequences. In total, 20 candidate genes were identified through blasting these sequences against the NCBI database (https://www.ncbi.nlm.nih.gov/). Three of the 47 original sequences were matched to *SlSnRK1* (Data S1; Bradford *et al.*, 2003; Avila *et al.*, 2012). The longest encodes the C-terminal 183 aa portion of SlSnRK1 (Supplementary Fig. S5A), and their interactions with SlSPL-CNR were further verified (Supplementary Fig. S5B, C). Moreover, we cloned the full-length SlSnRK1 coding sequence in-frame fused to the GAL4-activating and DNA-binding domain, as well as SlSPL-CNR in-frame fused to the GAL4 DNA binding domain (Supplementary Fig. S6). In two different configurations, the full-length SlSnRK1 protein was found to be able to interact with SlSPL-CNR (Fig. 6A, B).

**Fig. 7.**
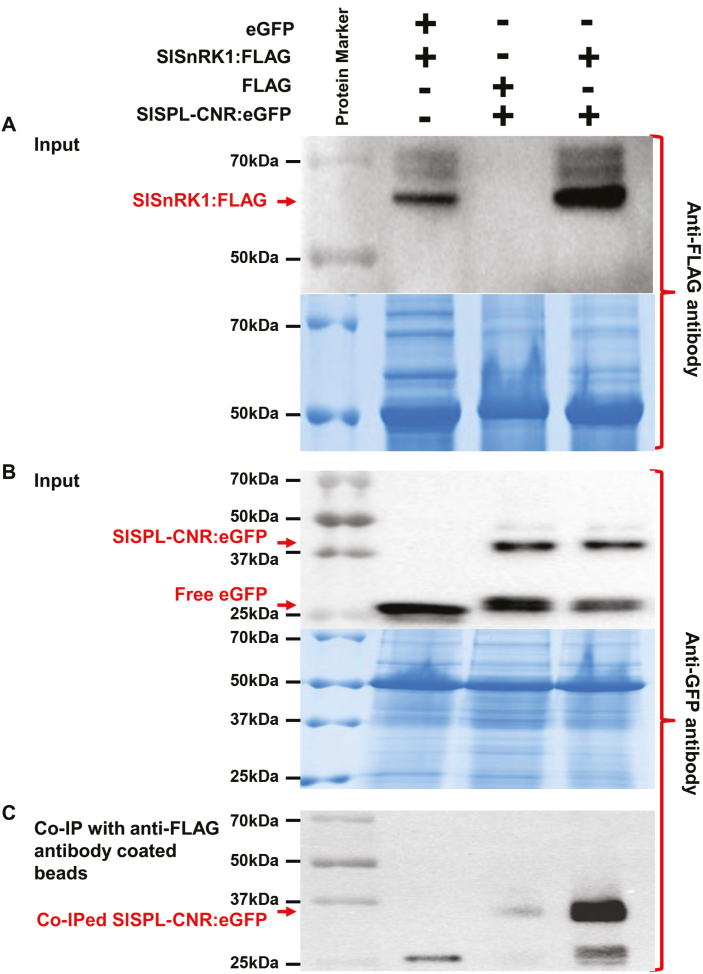
CoIP assays of interaction between SlSPL-CNR and SlSnRK1. (A, B) Detection of SlSPL-CNR:eGFP or SlSnRK1:FLAG in *N. benthamian* (Nb). Total proteins were extracted from Nb leaves at 3 d post-infiltration or co-infiltration with *A. tumefaciens* GV3101/pCAMBIA1300/35S-eGFP (eGFP) and GV3101/pCAMBIA1300/35S-SlSnRK1:FLAG (SlSnRK1:FLAG); GV3101/pCAMBIA1300/35S-FLAG (FLAG) and GV3101/pCAMBIA1300/35S-SlSPL-CNR:eGFP (SlSPL-CNR:eGFP); or GV3101/pCAMBIA1300/35S-SlSnRK1:FLAG and GV3101/pCAMBIA1300/35S-SlSPL-CNR:eGFP. Western blots were probed either with anti-3×FLAG antibody (A, upper panel) or anti-GFP antibody (B, upper panel). Positions for SlSnRK1:FLAG, SlSPL-CNR:eGFP fusion proteins as well as free eGFP are indicated by red arrows. Equal loading of protein samples was illustrated by Coomassie Blue staining gels (lower panel in (A, B)). (C) Detection of co-immunoprecipitated SlSPL-CNR:eGFP. Total proteins extracted from co-agroinfitrated Nb leaf tissues were absorbed with anti-FLAG^®^M2 Magnetic Beads, and analysed by western blot using anti-GFP antibody. Co-immunoprecipitation of SlSPL-CNR:eGFP by SlSnRK1:FLAG primarily occurred in leaf tissues co-infiltrated with GV3101/pCAMBIA1300/35S-SlSnRK1:FLAG and GV3101/pCAMBIA1300/35S-SlSPL-CNR:eGFP. The co-immunoprecipitated SlSPL-CNR:eGFP was readily detected by the anti-GFP antibody. The positions and sizes of protein marker are indicated.

Using a CoIP assay, we further examined if SlSPL-CNR interacts with SlSnRK1 in plants (Supplementary Fig. S7A–J; Fig. 7). Both SlSPL-CNR:eGFP (42 kDa) and SlSnRK1:FLAG (64 kDa) fusion proteins were readily detectable by either anti-GFP or anti-FLAG antibody (Supplementary Fig. S7K, L; Fig. 7A, B). SlSPL-CNR:eGFP was shown to be co-precipitated with SlSnRK1:FLAG (Fig. 7C). Moreover, expression of SlSPL-CNR:eGFP triggered cell death in agro-infiltrated tissues (Supplementary Fig. S7F, I, J), consistent with virus-transient expression assays. Collectively, our results clearly demonstrate that SlSPL-CNR can interact with SlSnRK1 in both yeast and plant cells.

### Silencing of SlSnRK1 inhibits tomato ripening

To investigate the biological relevance of the SlSPL-CNR/SlSnRK1 interaction in tomato, we first analysed *SlSnRK1* expression profiles in AC and *Cnr* fruits at various ripening stages and in different tissues (Supplementary Fig. S8). The qRT-PCR data indicate that expression of *SlSnRK1* underwent dynamic changes during fruit development and ripening (Supplementary Fig. S8A). Such oscillation in the *SlSnRK1* transcript levels from 30 to 45 DPA was particularly consistent with the RNA transcriptome (27–42 DPA) analysis (Supplementary Fig. S8B; original reads per kilobase of transcript per million mapped reads (RPKM) were from http://www.epigenome.cuhk.edu.hk/encode.html). Interestingly, the *SlSnRK1* mRNA level was slightly higher at most stages in *Cnr* than in AC fruits. This is in contrast to *SlSnRK1* being expressed more in AC stems, leaves, and flowers than in the equivalent *Cnr* tissues, whilst no difference was found in AC and *Cnr* roots (Supplementary Fig. S8C).

**Fig. 8.**
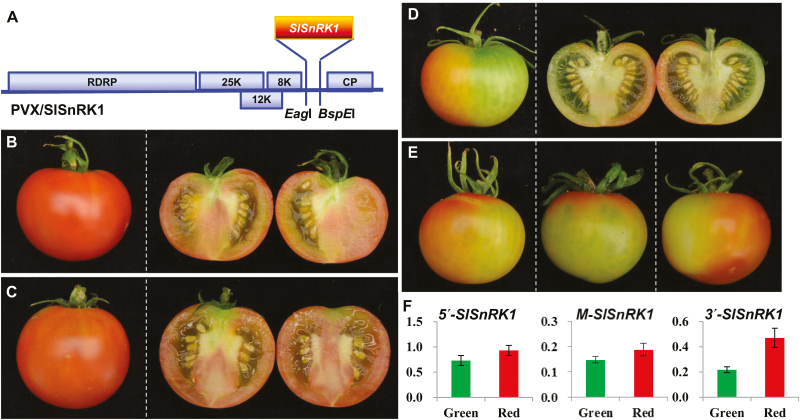
Silencing of *SlSnRK1* inhibits tomato fruit ripening. (A) Schematic representation of the VIGS vector PVX/SlSnRK1. Genome organization of PVX and the two cloning sites is indicated. RDRP is the viral RNA-dependent RNA polymerase. The triple-gene block encodes three viral movement proteins of 25, 12, and 8 kDa. CP is the viral coat protein. (B–E) VIGS of *SlSnRK1*. Mock-treated (B) and PVX-injected (C) AC fruits ripened. Fruits injected with PVX/SlSnRK1 developed non-ripe sectors (D, E). Fruits were photographed at 5 d after breaker (45 d post-anthesis). Fruits were cut in half to show ripe (B, C) or non-ripe (D) pericarps. Three more *SlSnRK1*-silenced AC fruits are shown in (E). (F) qRT-PCR analysis of *SlSnRK1* expression in *SlSnRK1*-silenced AC fruits. Expression of *SlSnRK1* was reduced by VIGS in non-ripe sectors (green bar) compared with the ripe sectors (red bar). qRT-PCRs were performed using three different sets of primers that target specific amplification of the 5′, middle (M), or 3′ end of the *SlSnRK1* gene (Supplementary Dataset S1). The relative levels (mean±SD) of the *SlSnRK1* transcripts against 18S rRNA differed among the three target RNA sequences, suggesting that VIGS efficiency as well as the transitivity of VIGS against the three portions of the *SlSnRK1* mRNA may be different. For each fruit we dissected the green non-ripe and red ripening sectors and extract total RNAs from each sectors. These RNAs were used in qRT-PCR assays along with three different sets of primers in order to examine how VIGS affected the level of SlSnRK1 mRNA transcripts. The relative expression level in the green or red sector of VIGSed fruits was further normalized against the level of SlSnRK1 mRNA in AC fruits at 40 d post-anthesis. Student’s *t*-test shows that the expression difference is of statistical significance (*P*=0.05). qRT-PCRs were performed on at least three different fruits and similar data were obtained for each fruit. Values in (F) are data generated from fruit shown in (D), normalized against the fruit in (B).

We then used VIGS to examine how *SlSnRK1* would affect fruit ripening (Fig. 8). To achieve this, pedicels of a total of 60–80 AC fruits at 5–20 DPA were mock-injected with Tris–EDTA buffer or injected with the empty VIGS vector PVX or PVX/SlSnRK1 (Fig. 8A; Supplementary Dataset S1). In all mock- or PVX-injected AC fruits, fruits developed and ripened normally (Fig. 8B, C). However, approximately 20% of AC fruits injected with PVX/SlSnRK1 showed delayed or non-ripening phenotypes (Fig. 8D, E), consistent with VIGS-mediated suppression of *SlSnRK1* gene expression in the non-ripe sectors of these fruits (Fig. 8F; Supplementary Fig. S9). It would be worthwhile mentioning that 20% of injected fruits showed phenotypes that are typical in our tomato VIGS experiments (Manning *et al.*, 2006; Lin *et al.*, 2008, Chen *et al.*, 2015*b*; Lai *et al.*, 2015).

To confirm the impact of *SlSnRK1* VIGS on tomato ripening, we analysed expression of a range of ripening-related genes in the green non-ripe and red-ripe sectors of the *SlSnRK1*-silenced AC fruits. These genes include key ripening TF genes, ethylene biosynthesis and responsive genes (Supplementary Fig. S10), and genes encoding enzymes for biosynthesis of lycopene, abscisic acid (ABA), carotenoids, and flavonoids (Supplementary Figs S11–S13). Consistent with the non-ripe phenotypes, expression levels of most of these genes were reduced in the non-ripe sectors compared with the red-ripe sectors in the *SlSnRK1*-silencing fruits. For instance, expression of *TDR4*, *RIN*, *NOR*, *NR*, and *SlSPL-CNR* was found to be markedly reduced in the non-ripe sectors. We also found a decrease in the expression level of ethylene biosynthesis and responsive genes such as *ACO1*, *ACO3*, *ACO4*, *ACS2*, *ACS3*, and *EBF2* (Supplementary Fig. S10). Similarly, expression levels of lycopene, carotenoid, and flavonoid biosynthesis genes including *PSY1*, *PSY2*, *PDS*, *ZDS*, *Z-ISO*, or *ANS* were reduced. The gene encoding the key enzyme 9-*cis*-epoxycarotenoid-dioxygenase for ABA biosynthesis was also decreased in the *SlSnRK1*-silenced non-ripe fruits (Supplementary Fig. S11).

### Differential methylation in the SlSnRK1 promoter

Compared with AC, *Cnr* fruit possesses a hypermethylated epigenome revealed by previously whole genome bisulfite sequencing studies in our and other laboratories (Zhong *et al.*, 2013; Chen *et al.*, 2015*b*). Using the latest tomato genome and epigenome databases, we analysed the DNA methylation profiles for *SlSnRK1*, particularly in the 5000-bp promoter sequences prior to the coding region (Supplementary Fig. S14). Two differentially methylated regions (DMRs) were identified in the *SlSnRK1* promoter. These DMRs were found to be highly methylated in *Cnr* compared with AC at 42 DPA. Interestingly, silencing of *SlCMT3*, which led to ripening reversion in *Cnr* fruits (Chen *et al.*, 2015*b*), reduced the DNA methylation level in both DMRs in the VIGS fruits compared with non-VIGS *Cnr* controls (Supplementary Fig. S14A, B). We interpret these results to mean that expression of *SlSnRK1*, similar to *SlSPL-CNR*, could be influenced by an epigenetic mechanism to affect fruit ripening in tomato.

**Fig. 9.**
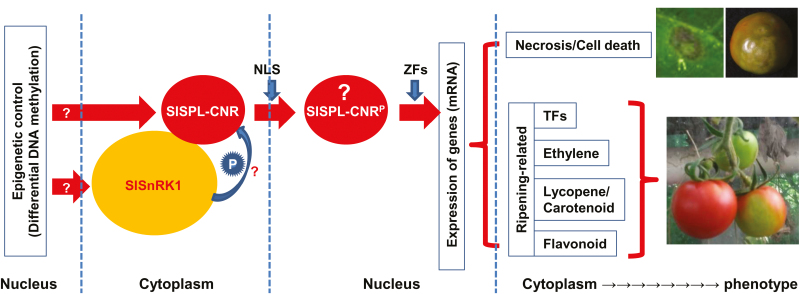
A working model of involvement of SlSnRK1 and SlSPL-CNR in cell death and fruit ripening in tomato. Epigenetic control may contribute to an extra layer of regulation of *SlSPL-CNR* and *SlSnRK1* (indicated by a question mark) expression in AC and *Cnr* tomato cell nucleus (Supplementary Fig. S14; Zhong *et al.*, 2013; Chen *et al.*, 2015b). SlSPL-CNR may undergo a post-translational phosphorylation in order to trigger its TF activity in the cytoplasm. Such cellular protein modification may be processed by SlSnRK1 through its direct interactions with the SlSPL-CNR protein (shown by a question mark). A phosphorylated SlSPL-CNR protein (designed SlSPL-CNR^**P**^, question mark) is then translocated via the unique monopartite NLS from the cytoplasm to the nucleus. However, phosphorylation *per se* may or may not be required for nuclear transportation of SlSPL-CNR^**P**^. Once located in the cell nucleus, SlSPL-CNR^**P**^ may bind to promoters in a zinc-dependent manner as for other SPB-box TFs to transcriptionally turn on or off expression of specific target genes associated with cell death and fruit ripening, which then leads to phenotypic induction of cell death and/or fruit ripening. Necrotic cell death on the tomato leaf and fruit as well as fruit with non-ripe sectors caused by either transient expression of SlSPL-CNR or virus induced gene silencing is shown. The leaf was photographed at 7 d post-inoculation and fruits at 40 d post-anthesis.

Comparative whole genome bisulfide sequencing analyses also imply that the *SlSnRK1* gene expression may be epigenetically affected. Expression of *SlSnRK1* occurred in fruits as well as other tissues in both AC and *Cnr*. This gene seems to be affected by *Cnr* (Supplementary Fig. S8), further suggesting that *SlSnRK1* may be influenced by an epigenetic mechanism and that SlSnRK1 may operate on SlSPL-CNR to affect fruit development and ripening. Interestingly, the levels of *SlSnRK1* mRNA in both AC and *Cnr* fruits are not that much different. It may be possible that in AC, the amount of SlSnRK1 protein translated from the limited amount of *SlSnRK1* transcripts might be sufficient to affect SlSPL-CNR function. On the other hand, higher levels of DNA methylation in *cis*-regulatory regions generally inhibit gene transcription. Nonetheless, single-base resolution profiling of whole tomato genome methylation along with transcriptome analysis has revealed many exceptions in which the opposite effects occur (Zhong *et al.*, 2013). It could be that higher methylation in the *cis*-differentially methylated sequences may block a repressor(s) to interact with the *SlSnRK1* promoter, resulting in a high level of *SlSnRK1* transcription in *Cnr*. However, any impact of SlSnRK1 on SlSPL-CNR in *Cnr* would be minimal due to the transcriptional blockage of *SlSPL-CNR* expression. Thus, these results may also imply that *SlSnRK1* may have an epistatic influence on *SlSPL-CNR*, presumably via a physical interaction between the two protein products, and subsequent phosphorylation of SlSPL-CNR by the kinase activity of SlSnRK1 (Fig. 9).

## Discussion

SlSPL-CNR has been shown to be involved in tomato fruit ripening. Suppression of *SlSPL-CNR* by an epimutation is responsible for the pleiotropic phenotypes in *Cnr* fruits (Thompson *et al.*, 1999; Eriksson *et al.*, 2004; Manning *et al.*, 2006). The *Cnr* epimutant also provides an important tool for investigating the (epi)genetic basis of tomato development and fruit ripening (Zhong *et al.*, 2013; Chen *et al.*, 2015*b*; Liu *et al.*, 2015). However, biochemical dissection of the SlSPL-CNR protein and the molecular mechanism for how this small TF affects tomato fruit ripening remain unknown. In this article, we report on the following discoveries.

First, SlSPL-CNR has a distinct NLS and is localized in the nucleus (Figs 1, 2). This unique NLS consists of ‘ _30_KRKR_33_’ at the N-terminus of SlSPL-CNR. Mutation of the monopartite NLS completely abolishes SlSPL-CNR localization in the nucleus despite the putative bipartite NLS at the C-terminus remaining intact (Fig. 2; Supplementary Fig. S1), differing from the bipartite NLS reported for other SBP-box TFs such as AtSPL3 and AtSPL8 (Birkenbihl *et al.*, 2005). Intriguingly, the monopartite NLS is unique so that no equivalent _30_KRKR_33_ signal sequence has been found in AtSPL3, AtSPL8, and other SBP-box TFs (Birkenbihl *et al.*, 2005).

Second, SlSPL-CNR is a zinc-binding protein that comprises two ZFMs, Zn1 and Zn2, within the C-terminal conserved SBP-box domain, and both ZFMs are involved in zinc binding (Fig. 4E; Supplementary Fig. S1). However, loss of Zn2 almost completely eliminates the zinc-binding activity of SlSPL-CNR. On the other hand, the Zn1-mutated SlSPL-CNR protein can still bind to zinc, albeit with a lower affinity than the wild-type protein (Fig. 4E). We observed that the intensity of the GFP fluorescence in plants expressing SlSPL-CNRmZn2:GFP or SlSPL-CNRmZn12:GFP was weaker than that found in plants expressing the wild-type SlSPL-CNR:GFP or SlSPL-CNRmZn1:GFP (Fig. 4A–D). This suggests that the Zn2 and Zn1/Zn2 mutants were less stable than the wild-type and Zn1 mutant SlSPL-CNR proteins in plant cells. Nevertheless, our findings are consistent with previous reports that both ZFMs are important for SBP-box TFs to bind to zinc and DNA in a zinc-dependent manner (Yamasaki *et al.*, 2004; Birkenbihl *et al.*, 2005).

Third, VIGC reveals that both NLS and ZFMs are functionally required for SlSPL-CNR to affect fruit ripening (Fig. 5), elucidating a previously unknown impact of NLS and ZFMs on SlSPL-CNR in tomato fruit ripening. It should be noted that during our VIGC experiments, we photographically recorded the change of these treated *Cnr* fruits. Partial complementation was well correlated with the viral transient expression of the wild-type *SlSPL-CNR* gene, but not with the any mutated forms of *SlSPL-CNR*, although the PVX coat protein could be detected in all these fruits (Fig. 5). From our experience, a change of fruit colour is a valid indication of fruit ripening, as shown in our previous work (Manning *et al.*, 2006, Lin *et al.*, 2008; Zhou *et al.* 2012; Chen *et al.*, 2015*a*,*b*, 2018*a*). In addition to its functionality in fruit ripening, SlSPL-CNR can also induce cell death in tomato and tobacco leaf tissues as well as in tomato fruits (Figs 1, 3, 4; Supplementary Fig. S7), indicating SlSPL-CNR is a multifunctional protein. Consistent with this idea, SlSPL-CNR was found to be expressed in leaves, early and late vegetative shoot apices, inflorescences, sepals, petals, and carpels although mainly in ripening fruits (Salinas *et al.*, 2012). Considering (i) transient expression of SlSPL-CNR via two means (i.e. virus- and aginfiltration-based vectors) caused cell death; (ii) NLS was required for SlSPL-CNR to induce cell death; and (iii) the two ZFMs were differentially involved in induction of cell death, we believe that activation of cell death is unlikely an artificial act for SlSPL-CNR. Moreover, viral ectopic expression of TFs does not always trigger cell death. For instance, LeMADS-RIN (SlMADS-RIN) when expressed from the same PVX-based vector caused no cell death, but resulted in VIGC (Zhou *et al.*, 2012). Another example is that viral expression of LeHB1 (SlHB1) initiated no cell death whilst disrupting flower development (Lin *et al.*, 2008). Both SlMADS-RIN and SlHB1 are two important ripening TFs in tomato. In addition, both stress-related genes and *DAD-1* encoding Defender against cell death-1 were also found to be up-regulated in *Cnr* (Eriksson *et al.*, 2004). Taken together, these different lines of evidence suggest that causing cell death is probably a genuine function of SlSPL-CNR along with its role in tomato ripening.

Fourth, in yeast and plant cells, SlSPL-CNR interacts with SlSnRK1 (Figs 6, 7; Supplementary Figs S4–S7). Moreover, the C-terminal 183 aa sequence of SlSnRK1 may have contributed to its interaction with SlSPL-CNR (Supplementary Fig. S5), although any precise interacting domain(s) needs to be further defined. To our knowledge, this is the first partner protein to be found to interact with SlSPL-CNR.

Fifth, suppression of *SlSnRK1* by VIGS inhibits fruit ripening and leads to reduction in the expression level of a wide range of ripening-related genes (Fig. 8; Supplementary Figs S9–S13). In these VIGSed AC fruits, only 20–30% reduction was observed in green sectors using the two sets of primers corresponding to the 5′ or middle portion of SlSnRK1. However, detection using a third pair of primers corresponding to the 3′ end of *SlSnRK1* showed more than 50% reduction of RNA transcript levels (Fig. 8F). These data indicate that the two sets of primers corresponding to the 5′ and middle parts of the gene likely picked up some untranslatable *SlSnRK1* mRNAs. Thus, the amount of *SlSnRK1* RNA detected in green portions might not be distinctively lower. It is also worth noting that the level of *SlSnRK1* mRNA tends to increase around breaker (35–37 DPA) and red-ripe stage (40 DPA) (Supplementary Fig. S8). These factors may contribute to a relatively low gene repression effect, yet a strong phenotype in these VIGSed AC fruits. Nevertheless, detections using all three sets of primers produced a very similar tendency of decreased *SlSnRK1* levels in green sectors when compared with red sectors.

Together, these collective findings suggest that *SlSnRK1* transcription and subsequent post-translational SlSPL-CNR–SlSnRK1 interaction are of biological relevance to tomato fruit ripening (Supplementary Fig. S14; Fig. 9). Indeed, VIGS experiments revealed that *SlSnRK1* is involved in fruit ripening. Our working model (Fig. 9) suggests that involvement of *SlSnRK1* in fruit ripening might be via the physical protein interaction between the *SlSnRK1* gene product and SlSPL-CNR, and subsequent phosphorylation of SlSPL-CNR by the kinase activity of SlSnRK1. Such phosphorylation of SlSPL-CNR by SlSnRK1 is supposed to occur in the cytoplasm. Translocation of phosphorylated SlSPL-CNR from the cytoplasm to the nucleus is mainly determined by the unique monopartite NLS. However, a potential requirement of phosphorylation of SlSPL-CNR for its transfer to the nucleus is also possible. We are now trying to design experiments to test if phosphorylation of SlSPL-CNR by SlSnRK1 occurs, and if interfering with this process would interrupt nuclear localization of CNR, cell death, and ripening as predicted by our working model.

Interestingly, transgenic overexpression of a heterologous *MhSnRK1* gene isolated from *Malus hupehensis* was reported to increase carbon assimilation and nitrogen uptake in tomato. Moreover, fruits expand faster at the early stage of development after anthesis and fruit-set, and reach the breaker/colour-turning point earlier in the *MhSnRK1* transgenic tomato plants compared with non-transgenic controls (Wang *et al.*, 2012). These findings suggest that *MhSnRK1* may act as a facilitator for fruit ripening in the transgenic plants, consistent with suppression of fruit ripening by *SlSnRK1* VIGS (this study). However, in strawberry (*Fragaria* × *ananassa*), FaSnRK2 has been found to interact with ABSCISIC ACID INSENSITIVE1, a negative regulator of fruit ripening. RNAi of *FaSnRK2* significantly promotes whilst overexpression of *FaSnRK2* arrests ripening, demonstrating that *FaSnRK2* negatively impacts on fruit ripening in strawberry (Han *et al.*, 2015). These observations may suggest complex and different functions of SnRKs in climacteric and non-climacteric fruit ripening. Moreover, *SnRK1* family genes including *SlSnRK1* have been found in response to biotic and abiotic stress, cell death, and hypersensitive response in tomato and a wide range of plants (Szczesny *et al.*, 2010; Cho *et al.*, 2012; Avila *et al.*, 2012; Lin *et al.*, 2014; Perochon *et al.*, 2015). It is thus possible that the SlSPL-CNR–SlSnRK1 interactions may be also required for induction of necrosis in plants.

Recently, using the CRISPR/Cas9 gene editing technique, Gao *et al.* (2019) produced *Cnr* and *nor* knockout mutants whilst Wang *et al.* (2019) generated null mutants for *ap2a*, *nor*, and *ful1/2* in order to re-evaluate functions of these TFs in tomato development and fruit ripening. Interestingly, the bioengineered *ap2a* null mutants produced delayed ripening fruits similar to those in RNAi lines (Wang *et al.*, 2019). However, CRSIPR/Cas9 knockout mutants for *Cnr*, *nor*, and *ful1*/2 all failed to phenocopy non-ripening as seen in each of the naturally occurring mutants or in RNAi or VIGS fruits (Gao *et al.*, 2019; Wang *et al.*, 2019). Such phenotypic discrepancies raise an intriguing issue about the precise functionality of the four TFs in tomato fruit ripening. Different hypotheses such as dominant-negative protein, gain-of-function, overlapping functions, and functional redundancy have been put forward in order to explain how CNR, NOR, and FUL1/FUL2 act in tomato ripening. On the other hand, genetically engineered knockout mutants of genes essential for development do not often show any obvious phenotype as shown in naturally occurring mutants or in silencing/RNAi-based knockdown lines. This phenomenon is not uncommon and has been well studied in animals, although seldom reported in plants. It could be explained by genetic compensation, more specifically, transcriptional adaptation that has been shown to be triggered by nonsense mutated mRNA degradation in mice and zebrafish (El-Brolosy *et al.*, 2019; Ma *et al.*, 2019). By analogy, the tomato knockouts versus knockdown/natural mutants may represent rare examples of genetic compensation in plants, reinforcing that TFs such as SlSPL-CNR, NOR, and FUL1/2 may play essential roles not only in fruit ripening but also in other physiological processes.

## Summary

We report that the SlSPL-CNR protein, an SBP-box TF, can affect tomato fruit ripening and cause cell death in tomato and tobacco plants. Considering the enzymatic activities of SlSnRK1 in phosphorylation of proteins, we envisage a working model that may provide a plausible explanation about how SlSPL-CNR functions as a multi-functional protein to activate tomato fruit ripening and to trigger plant cell death (Fig. 9). We propose that SlSPL-CNR might be post-translationally phosphorylated by SlSnRK1 through direct physical interactions in the cytoplasm. Indeed, SlSnRK1 has been shown to have protein phosphorylation activity (Su and Devarenne, 2018) and it can phosphorylate its interacting partner in tomato (Shen *et al.*, 2011). Thus, a phosphorylated SlSPL-CNR protein might be then translocated from the cytoplasm to the nucleus, which is mainly determined via the unique monopartite NLS. Once located in the cell nucleus, SlSPL-CNR might bind to promoters in a zinc-dependent manner to turn on or off expression of target genes associated with cell death and fruit ripening.

## Supplementary data

Supplementary data are available at *JXB* online.

Dataset S1. *SlSnRK1* sequence and primers used for construction of its VIGS vector and qRT-PCRs.

Fig. S1. SlSPL-CNR and Arabidopsis AtSPL3 sequences and amino-acid domains.

Fig. S2. PVX-based gene expression vectors for characterizing the SlSPL-CNR NLS and VIGC.

Fig. S3. PVX-based gene expression vectors for characterizing the SlSPL-CNR ZFMs and VIGC.

Fig. S4. Yeast-two-hybrid screening of SlSPL-CNR interacting proteins.

Fig. S5. Yeast-two-hybrid screening identifies three partial sequences coding for SlSnRK1 polypeptides that interact with SlSPL-CNR.

Fig. S6. Construction of SlSnRK1 and SlSPL-CNR full-length gene expression vectors for Y2H protein–protein interaction assay.

Fig. S7. CoIP analysis of SlSPL-CNR and SlSnRK1 protein interaction.

Fig. S8 *SlSnRK1* expression in tomato.

Fig. S9. Detection of PVX/SlSnRK1 in tomato fruits.

Fig. S10. qRT-PCR analyses of ripening-related TF and ethylene biosynthesis and responsive genes in non-ripe and ripe sectors of *SlSnRK1*-silenced AC fruits.

Fig. S11. qRT-PCR analyses of expression of lycopene, ABA, carotenoid, and flavonoid biosynthesis and other ripening-related genes in non-ripe and ripe sectors of *SlSnRK1-*silenced AC fruits.

Fig. S12. Genes involved in lycopene, carotenoid, and abscisic acid (ABA) biosynthesis.

Fig. S13. Genes involved in flavonoid biosynthesis.

Fig. S14. DNA methylation profiles for the *SlSnRK1* gene.

Table S1. Summary of cellular localization of wild-type and mutant SlSPL-CNRs and their functionality to induce cell death.

Table S2. Primers used for constructions of SlSPL-CNR NLS and ZFM mutants.

Table S3. Primers used for qRT-PCR.

Table S4. Additional primers and their use for construction of gene expression cassettes.

eraa067_suppl_supplementary_figures_S1_S14

eraa067_suppl_supplementary_tables_S1_S4

eraa067_suppl_supplementary_dataset_S1

## Abbreviations

CoIP: co-immunoprecipitation
Cnr: Colourless non-ripening
GFP: green fluorescent protein
NLS: nuclear localization signal
PVX: potato virus X
Sl: *Solanum lycopersicum*
SlCMT2: CHROMOMETHYLASE 2
SlDML2: DEMETER-like DNA demethylase 2
SlDRM7: DOMAINS REARRANGED METHYLTRANSFERASE 7
SlMET1: METHYLTRANSFERASE 1
SlSnRK1: SUCROSE NONFERMENTING1-RELATED PROTEIN KINASE1
SNF1: SUCROSE NONFERMENTING1
SPL: SQUAMOSA Promoter Binding Protein-like
TF: transcription factor
VIGC: virus-induced gene complementation
VIGS: virus-induced gene silencing
Y2H: yeast two-hybrid
ZFM: zinc-finger motif

## References

[CIT0001] Avila J, Gregory OG, Su D, Deeter TA, Chen S, Silva-Sanchez C, Xu S, Martin GB, Devarenne TP 2012 The β-subunit of the SnRK1 complex is phosphorylated by the plant cell death suppressor Adi3. Plant Physiology 159, 1277–1290. 22573803 10.1104/pp.112.198432 PMC3387709

[CIT0002] Birkenbihl RP, Jach G, Saedler H, Huijser P 2005 Functional dissection of the plant-specific SBP-domain: overlap of the DNA-binding and nuclear localization domains. Journal of Molecular Biology 352, 585–596. 16095614 10.1016/j.jmb.2005.07.013

[CIT0003] Bledsoe SW, Henry C, Griffiths CA, Paul MJ, Feil R, Lunn JE, Stitt M, Lagrimini LM 2017 The role of Tre6P and SnRK1 in maize early kernel development and events leading to stress-induced kernel abortion. BMC Plant Biology 17, 74. 28403831 10.1186/s12870-017-1018-2 PMC5389189

[CIT0004] Bradford KJ, Downie AB, Gee OH, Alvarado V, Yang H, Dahal P 2003 Abscisic acid and gibberellin differentially regulate expression of genes of the SNF1-related kinase complex in tomato seeds. Plant Physiology 132, 1560–1576. 12857836 10.1104/pp.102.019141 PMC167094

[CIT0005] Cardon G, Höhmann S, Klein J, Nettesheim K, Saedler H, Huijser P 1999 Molecular characterisation of the *Arabidopsis* SBP-box genes. Gene 237, 91–104. 10524240 10.1016/s0378-1119(99)00308-x

[CIT0006] Cardon GH, Höhmann S, Nettesheim K, Saedler H, Huijser P 1997 Functional analysis of the *Arabidopsis thaliana* SBP-box gene *SPL3*: a novel gene involved in the floral transition. The Stand Journal 12, 367–377. 10.1046/j.1365-313x.1997.12020367.x 9301089

[CIT0007] Chen W, Kong J, Lai T, et al. 2015 *a* Tuning *LeSPL-CNR* expression by SlymiR157 affects tomato fruit ripening. Scientific Reports 5, 7852. 25597857 10.1038/srep07852 PMC4297963

[CIT0008] Chen W, Kong J, Qin C, et al. 2015 *b* Requirement of *CHROMOMETHYLASE3* for somatic inheritance of the spontaneous tomato epimutation *Colourless non-ripening*. Scientific Reports 5, 9192. 25778911 10.1038/srep09192 PMC4361866

[CIT0009] Chen W, Yu Z, Kong J, et al. 2018 *a* Comparative WGBS identifies genes that influence non-ripe phenotype in tomato epimutant *Colourless non-ripening*. Science China. Life Sciences 61, 244–252. 29288427 10.1007/s11427-017-9206-5

[CIT0010] Chen W, Zhang X, Fan Y, et al. 2018 *b* A genetic network for systemic RNA silencing in plants. Plant Physiology 176, 2700–2719. 29439213 10.1104/pp.17.01828 PMC5884585

[CIT0011] Cho YH, Hong JW, Kim EC, Yoo SD 2012 Regulatory functions of SnRK1 in stress-responsive gene expression and in plant growth and development. Plant Physiology 158, 1955–1964. 22232383 10.1104/pp.111.189829 PMC3320198

[CIT0012] Chuck GS, Brown PJ, Meeley R, Hake S 2014 Maize SBP-box transcription factors unbranched2 and unbranched3 affect yield traits by regulating the rate of lateral primordia initiation. Proceedings of the National Academy of Sciences, USA 111, 18775–18780. 10.1073/pnas.1407401112 PMC4284592 25512525

[CIT0013] Chuck G, Whipple C, Jackson D, Hake S 2010 The maize SBP-box transcription factor encoded by tasselsheath4 regulates bract development and the establishment of meristem boundaries. Development 137, 1243–1250. 20223762 10.1242/dev.048348

[CIT0014] Coello P, Hey SJ, Halford NG 2011 The sucrose non-fermenting-1-related (SnRK) family of protein kinases: potential for manipulation to improve stress tolerance and increase yield. Journal of Experimental Botany 62, 883–893. 20974737 10.1093/jxb/erq331

[CIT0015] El-Brolosy MA, Kontarakis Z, Rossi A, et al. 2019 Genetic compensation triggered by mutant mRNA degradation. Nature 568, 193–197. 30944477 10.1038/s41586-019-1064-z PMC6707827

[CIT0016] Eriksson EM, Bovy A, Manning K, Harrison L, Andrews J, De Silva J, Tucker GA, Seymour GB 2004 Effect of the colorless non-ripening mutation on cell wall biochemistry and gene expression during tomato fruit development and ripening. Plant Physiology 136, 4184–4197. 15563627 10.1104/pp.104.045765 PMC535848

[CIT0017] Ferreira e Silva GF, Silva EM, Azevedo Mda S, Guivin MA, Ramiro DA, Figueiredo CR, Carrer H, Peres LE, Nogueira FT 2014 microRNA156-targeted SPL/SBP box transcription factors regulate tomato ovary and fruit development. Plant Journal 78, 604–618. 24580734 10.1111/tpj.12493

[CIT0018] Gao XQ, Liu CZ, Li DD, Zhao TT, Li F, Jia XN, Zhao XY, Zhang XS 2016 The *Arabidopsis* KINβγ subunit of the SnRK1 complex regulates pollen hydration on the stigma by mediating the level of reactive oxygen species in pollen. PLoS Genetics 12, e1006228. 27472382 10.1371/journal.pgen.1006228 PMC4966946

[CIT0019] Gao Y, Zhu N, Zhu X, et al. 2019 Diversity and redundancy of the ripening regulatory networks revealed by the fruitENCODE and the new CRISPR/Cas9 CNR and NOR mutants. Horticulture Research 6, 39. 30774962 10.1038/s41438-019-0122-x PMC6370854

[CIT0020] Guérinier T, Millan L, Crozet P, et al. 2013 Phosphorylation of p27(KIP1) homologs KRP6 and 7 by SNF1-related protein kinase-1 links plant energy homeostasis and cell proliferation. The Plant Journal 75, 515–525. 23617622 10.1111/tpj.12218

[CIT0021] Han Y, Dang R, Li J, Jiang J, Zhang N, Jia M, Wei L, Li Z, Li B, Jia W 2015 SUCROSE NONFERMENTING1-RELATED PROTEIN KINASE2.6, an ortholog of OPEN STOMATA1, is a negative regulator of strawberry fruit development and ripening. Plant Physiology 167, 915–930. 25609556 10.1104/pp.114.251314 PMC4348756

[CIT0022] Hou H, Yan X, Sha T, Yan Q, Wang X 2017 The SBP-box gene *VpSBP11* from Chinese wild *Vitis* is involved in floral transition and affects leaf development. International Journal of Molecular Sciences 18, E1493. 28703739 10.3390/ijms18071493 PMC5535983

[CIT0023] Huijser P, Klein J, Lönnig WE, Meijer H, Saedler H, Sommer H 1992 Bracteomania, an inflorescence anomaly, is caused by the loss of function of the MADS-box gene *SQUAMOSA* in *Antirrhinum majus*. The EMBO Journal 11, 1239–1249. 1563342 10.1002/j.1460-2075.1992.tb05168.x PMC556572

[CIT0024] Kim GD, Cho YH, Yoo SD 2017 Regulatory functions of cellular energy sensor SNF1-related Kinase1 for leaf senescence delay through *ETHYLENE-INSENSITIVE3* repression. Scientific Reports 7, 3193. 28600557 10.1038/s41598-017-03506-1 PMC5466610

[CIT0025] Klein J, Saedler H, Huijser P 1996 A new family of DNA-binding proteins includes putative transcriptional regulators of the *Antirrhinum majus* floral meristem identity genes *SAUAMOSA*. Molecular and General Genetics 259, 7–16. 10.1007/BF02191820 8569690

[CIT0026] Kong J, Chen W, Shen J, Qin C, Lai T, Zhang P, Wang Y, Wu C, Yang X, Hong Y 2013 Virus-induced gene complementation in tomato. Plant Signaling & Behavior 8, e27142. 24305652 10.4161/psb.27142 PMC4091552

[CIT0027] Lai T, Wang Y, Zhou T, Mei F, Zhang P, Zhou Y, Shi N, Hong Y 2015 Virus-induced *LeSPL-CNR* silencing inhibits fruit ripening in tomato. Journal of Agricultural Sciences 7, 184–194.

[CIT0028] Lawlor DW, Paul MJ 2014 Source/sink interactions underpin crop yield: the case for trehalose 6-phosphate/SnRK1 in improvement of wheat. Frontiers in Plant Science 5, 418. 25202319 10.3389/fpls.2014.00418 PMC4142875

[CIT0029] Li C, Gu M, Shi N, et al. 2011 Mobile FT mRNA contributes to the systemic florigen signalling in floral induction. Scientific Reports 1, 73. 22355592 10.1038/srep00073 PMC3216560

[CIT0030] Lin CR, Lee KW, Chen CY, Hong YF, Chen JL, Lu CA, Chen KT, Ho TH, Yu SM 2014 SnRK1A-interacting negative regulators modulate the nutrient starvation signaling sensor SnRK1 in source-sink communication in cereal seedlings under abiotic stress. The Plant Cell 26, 808–827. 24569770 10.1105/tpc.113.121939 PMC3967042

[CIT0031] Lin Z, Hong Y, Yin M, Li C, Zhang K, Grierson D 2008 A tomato HD-Zip homeobox protein, LeHB-1, plays an important role in floral organogenesis and ripening. The Plant Journal 55, 301–310. 18397374 10.1111/j.1365-313X.2008.03505.x PMC2607530

[CIT0032] Liu R, How-Kit A, Stammitti L, et al. 2015 A DEMETER-like DNA demethylase governs tomato fruit ripening. Proceedings of the National Academy of Sciences, USA 112, 10804–10809. 10.1073/pnas.1503362112 PMC4553810 26261318

[CIT0033] Liu XJ, An XH, Liu X, Hu DG, Wang XF, You CX, Hao YJ 2017 MdSnRK1.1 interacts with MdJAZ18 to regulate sucrose-induced anthocyanin and proanthocyanidin accumulation in apple. Journal of Experimental Botany 68, 2977–2990. 28549152 10.1093/jxb/erx150 PMC5853841

[CIT0034] Livak KJ, Schmittgen TD 2001 Analysis of relative gene expression data using real-time quantitative PCR and the 2^−^^ΔΔ*C*(T)^ method. Methods 25, 402–408. 10.1006/meth.2001.1262 11846609

[CIT0035] Lu CA, Lin CC, Lee KW, Chen JL, Huang LF, Ho SL, Liu HJ, Hsing YI, Yu SM 2007 The SnRK1A protein kinase plays a key role in sugar signaling during germination and seedling growth of rice. The Plant Cell 19, 2484–2499. 17766403 10.1105/tpc.105.037887 PMC2002608

[CIT0036] Ma Z, Zhu P, Shi H, Guo L, Zhang Q, Chen Y, Chen S, Zhang Z, Peng J, Chen J 2019 PTC-bearing mRNA elicits a genetic compensation response via Upf3a and COMPASS components. Nature 568, 259–263. 30944473 10.1038/s41586-019-1057-y

[CIT0037] Manning K, Tör M, Poole M, Hong Y, Thompson AJ, King GJ, Giovannoni JJ, Seymour GB 2006 A naturally occurring epigenetic mutation in a gene encoding an SBP-box transcription factor inhibits tomato fruit ripening. Nature Genetics 38, 948–952. 16832354 10.1038/ng1841

[CIT0038] Perochon A, Jianguang J, Kahla A, Arunachalam C, Scofield SR, Bowden S, Wallington E, Doohan FM 2015 *TaFROG* encodes a pooideae orphan protein that interacts with SnRK1 and enhances resistance to the mycotoxigenic fungus *Fusarium graminearum*. Plant Physiology 169, 2895–2906. 26508775 10.1104/pp.15.01056 PMC4677899

[CIT0039] Preston JC, Hileman LC 2010 *SQUAMOSA*-PROMOTER BINDING PROTEIN 1 initiates flowering in *Antirrhinum majus* through the activation of meristem identity genes. The Plant Journal 62, 704–712. 20202170 10.1111/j.1365-313X.2010.04184.x

[CIT0040] Qin C, Chen W, Shen J, et al. 2017 A virus-induced assay for functional dissection and analysis of monocot and dicot flowering time genes. Plant Physiology 174, 875–885. 28400493 10.1104/pp.17.00392 PMC5462034

[CIT0041] Qin C, Shi N, Gu M, et al. 2012 Involvement of *RDR6* in short-range intercellular RNA silencing in *Nicotiana benthamiana*. Scientific Reports 2, 467. 22737403 10.1038/srep00467 PMC3381291

[CIT0042] Salinas M, Xing S, Höhmann S, Berndtgen R, Huijser P 2012 Genomic organization, phylogenetic comparison and differential expression of the SBP-box family of transcription factors in tomato. Planta 235, 1171–1184. 22160465 10.1007/s00425-011-1565-y

[CIT0043] Schwachtje J, Minchin PE, Jahnke S, van Dongen JT, Schittko U, Baldwin IT 2006 SNF1-related kinases allow plants to tolerate herbivory by allocating carbon to roots. Proceedings of the National Academy of Sciences, USA 103, 12935–12940. 10.1073/pnas.0602316103 PMC1568949 16912118

[CIT0044] Schwarz S, Grande AV, Bujdoso N, Saedler H, Huijser P 2008 The microRNA regulated SBP-box genes *SPL9* and *SPL15* control shoot maturation in *Arabidopsis*. Plant Molecular Biology 67, 183–195. 18278578 10.1007/s11103-008-9310-z PMC2295252

[CIT0045] Shen Q, Liu Z, Song F, Xie Q, Hanley-Bowdoin L, Zhou X 2011 Tomato SlSnRK1 protein interacts with and phosphorylates βC1, a pathogenesis protein encoded by a geminivirus β-satellite. Plant Physiology 157, 1394–1406. 21885668 10.1104/pp.111.184648 PMC3252149

[CIT0046] Shikata M, Koyama T, Mitsuda N, Ohme-Takagi M 2009 *Arabidopsis* SBP-box genes *SPL10*, *SPL11* and *SPL2* control morphological change in association with shoot maturation in the reproductive phase. Plant & Cell Physiology 50, 2133–2145. 19880401 10.1093/pcp/pcp148

[CIT0047] Stone JM, Liang X, Nekl ER, Stiers JJ 2005 *Arabidopsis AtSPL14*, a plant-specific SBP-domain transcription factor, participates in plant development and sensitivity to fumonisin B1. The Plant Journal 41, 744–754. 15703061 10.1111/j.1365-313X.2005.02334.x

[CIT0048] Su D, Devarenne TP 2018 In vitro activity characterization of the tomato SnRK1 complex proteins. Biochimica et Biophysica Acta. Proteins and Proteomics 1866, 857–864. 29777861 10.1016/j.bbapap.2018.05.010

[CIT0049] Szczesny R, Büttner D, Escolar L, Schulze S, Seiferth A, Bonas U 2010 Suppression of the AvrBs1-specific hypersensitive response by the YopJ effector homolog AvrBsT from *Xanthomonas* depends on a SNF1-related kinase. New Phytologist 187, 1058–1074. 20609114 10.1111/j.1469-8137.2010.03346.x

[CIT0050] Thompson AJ, Tor M, Barry CS, Vrebalov J, Orfila C, Jarvis MC, Giovannoni JJ, Grierson D, Seymour GB 1999 Molecular and genetic characterization of a novel pleiotropic tomato-ripening mutant. Plant Physiology 120, 383–390. 10364389 10.1104/pp.120.2.383 PMC59276

[CIT0051] Unte US, Sorensen AM, Pesaresi P, Gandikota M, Leister D, Saedler H, Huijser P 2003 *SPL8*, an SBP-box gene that affects pollen sac development in Arabidopsis. The Plant Cell 15, 1009–1019. 12671094 10.1105/tpc.010678 PMC152345

[CIT0052] van Wezel R, Hong Y 2004 Virus survival of RNA silencing without deploying protein-mediated suppression in *Nicotiana benthamiana*. FEBS Letters 562, 65–70. 15044003 10.1016/S0014-5793(04)00184-X

[CIT0053] van Wezel R, Liu H, Wu Z, Stanley J, Hong Y 2003 Contribution of the zinc finger to zinc and DNA binding by a suppressor of posttranscriptional gene silencing. Journal of Virology 77, 696–700. 12477872 10.1128/JVI.77.1.696-700.2003 PMC140617

[CIT0054] van Wezel R, Dong X, Liu H, Tien P, Stanley J, Hong Y 2002 Mutation of three cysteine residues in Tomato yellow leaf curl virus-China C2 protein causes dysfunction in pathogenesis and posttranscriptional gene-silencing suppression. Molecular Plant-Microbe Interactions 15, 203–208. 11952122 10.1094/MPMI.2002.15.3.203

[CIT0055] Wang H, Nussbaum-Wagler T, Li B, Zhao Q, Vigouroux Y, Faller M, Bomblies K, Lukens L, Doebley JF 2005 The origin of the naked grains of maize. Nature 436, 714–719. 16079849 10.1038/nature03863 PMC1464477

[CIT0056] Wang L, Zhang Q 2017 Boosting rice yield by fine-tuning SPL gene expression. Trends in Plant Science 22, 643–646. 28647102 10.1016/j.tplants.2017.06.004

[CIT0057] Wang R, da Rocha Tavano EC, Lammers M, Martinelli AP, Angenent GC, de Maagd RA 2019 Re-evaluation of transcription factor function in tomato fruit development and ripening with CRISPR/Cas9-mutagenesis. Scientific Reports 9, 1696. 30737425 10.1038/s41598-018-38170-6 PMC6368595

[CIT0058] Wang X, Peng F, Li M, Yang L, Li G 2012 Expression of a heterologous SnRK1 in tomato increases carbon assimilation, nitrogen uptake and modifies fruit development. Journal of Plant Physiology 169, 1173–1182. 22727046 10.1016/j.jplph.2012.04.013

[CIT0059] Wu X, Li Y, Shi Y, Song Y, Zhang D, Li C, Buckler ES, Li Y, Zhang Z, Wang T 2016 *a* Joint-linkage mapping and GWAS reveal extensive genetic loci that regulate male inflorescence size in maize. Plant Biotechnology Journal 14, 1551–1562. 26801971 10.1111/pbi.12519 PMC5066742

[CIT0060] Wu Z, Cao Y, Yang R, Qi T, Hang Y, Lin H, Zhou G, Wang ZY, Fu C 2016 *b* Switchgrass SBP-box transcription factors PvSPL1 and 2 function redundantly to initiate side tillers and affect biomass yield of energy crop. Biotechnology for Biofuels 9, 101. 27158262 10.1186/s13068-016-0516-z PMC4858904

[CIT0061] Xing S, Salinas M, Garcia-Molina A, Höhmann S, Berndtgen R, Huijser P 2013 *SPL8* and miR156-targeted *SPL* genes redundantly regulate Arabidopsis gynoecium differential patterning. The Plant Journal 75, 566–577. 23621152 10.1111/tpj.12221

[CIT0062] Xing S, Salinas M, Höhmann S, Berndtgen R, Huijser P 2010 miR156-targeted and nontargeted SBP-box transcription factors act in concert to secure male fertility in *Arabidopsis*. The Plant Cell 22, 3935–3950. 21177480 10.1105/tpc.110.079343 PMC3027167

[CIT0063] Yamaguchi A, Wu MF, Yang L, Wu G, Poethig RS, Wagner D 2009 The microRNA-regulated SBP-Box transcription factor SPL3 is a direct upstream activator of LEAFY, FRUITFULL, and APETALA1. Developmental Cell 17, 268–278. 19686687 10.1016/j.devcel.2009.06.007 PMC2908246

[CIT0064] Yamasaki K, Kigawa T, Inoue M, et al. 2004 A novel zinc-binding motif revealed by solution structures of DNA-binding domains of *Arabidopsis* SBP-family transcription factors. Journal of Molecular Biology 337, 49–63. 15001351 10.1016/j.jmb.2004.01.015

[CIT0065] Yu Z, Chen Q, Chen W, et al. 2018 Multigene editing via CRISPR/Cas9 guided by a single-sgRNA seed in *Arabidopsis*. Journal of Integrative Plant Biology 60, 376–381. 29226588 10.1111/jipb.12622

[CIT0066] Zhang B, Liu X, Zhao G, Mao X, Li A, Jing R 2014 Molecular characterization and expression analysis of *Triticum aestivum* squamosa-promoter binding protein-box genes involved in ear development. Journal of Integrative Plant Biology 56, 571–581. 24386921 10.1111/jipb.12153 PMC4239008

[CIT0067] Zhang B, Xu W, Liu X, Mao X, Li A, Wang J, Chang X, Zhang X, Jing R 2017 Functional conservation and divergence among homoeologs of *TaSPL20* and *TaSPL21*, two SBP-box genes governing yield-related traits in hexaploid wheat. Plant Physiology 174, 1177–1191. 28424214 10.1104/pp.17.00113 PMC5462027

[CIT0068] Zhang SD, Ling LZ, Yi TS 2015 Evolution and divergence of SBP-box genes in land plants. BMC Genomics 16, 787. 26467431 10.1186/s12864-015-1998-y PMC4606839

[CIT0069] Zhang Y, Shewry PR, Jones H, Barcelo P, Lazzeri PA, Halford NG 2001 Expression of antisense SnRK1 protein kinase sequence causes abnormal pollen development and male sterility in transgenic barley. The Plant Journal 28, 431–441. 11737780 10.1046/j.1365-313x.2001.01167.x

[CIT0070] Zhong S, Fei Z, Chen YR, et al. 2013 Single-base resolution methylomes of tomato fruit development reveal epigenome modifications associated with ripening. Nature Biotechnology 31, 154–159. 10.1038/nbt.2462 23354102

[CIT0071] Zhou T, Zhang H, Lai T, et al. 2012 Virus-induced gene complementation reveals a transcription factor network in modulation of tomato fruit ripening. Scientific Reports 2, 836. 23150786 10.1038/srep00836 PMC3495281

