# Supplementary material for: Molecular and functional characterization of the SBP-box transcription factor SPL-CNR in tomato fruit ripening and cell death
Source: J Exp Bot. 2020 Feb 4;71(10):2995–3011. doi: 10.1093/jxb/eraa067 (PMC7260717; doi:10.1093/jxb/eraa067)
Supplement: eraa067_suppl_supplementary_figures_S1_S14 [file eraa067_suppl_supplementary_figures_s1_s14.pdf]

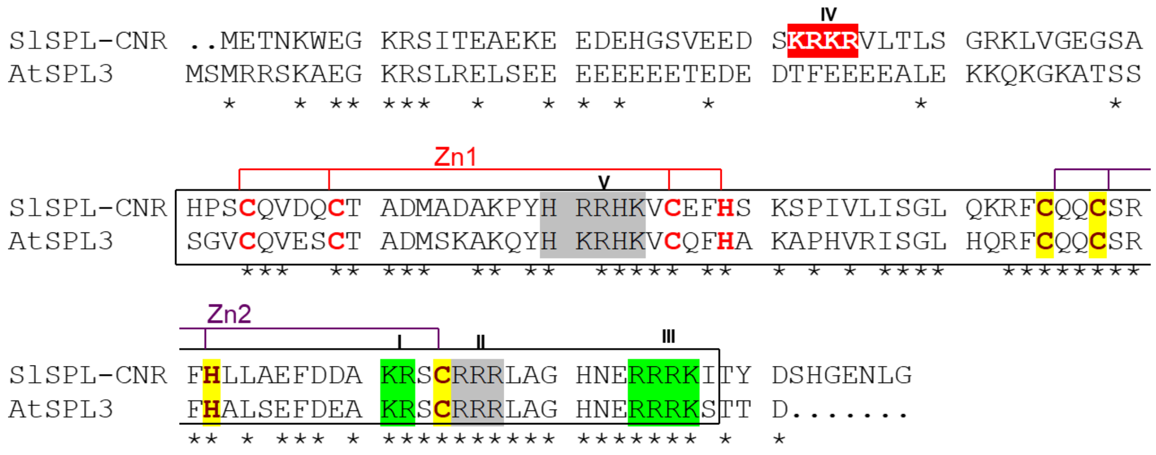

Fig. S1 SlSPL-CNR and Arabidopsis AtSPL3 sequences and amino-acid domains. Identical amino acids are marked by asterisks. The conserved SBP-box domain is boxed. Five basic amino acid domains I-V are highlighted as indicated. Domains I and III (highlighted green) are supposed to be the nuclear localization signal for AtSPL3. Domain IV (highlighted red) is unique to SlSPL-CNR. Two putative zinc-finger motifs (ZFMs) are indicated as Zn1 and Zn2.

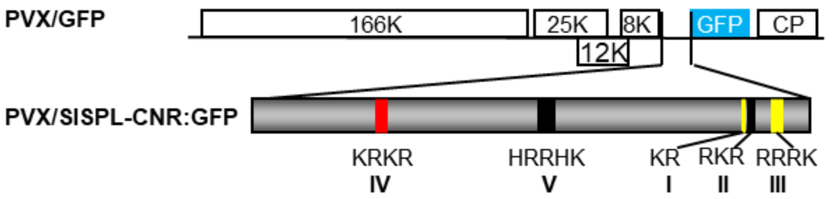

	IV	V	I	II	III
Single-domain mutants					
PVX/SlSPL-CNR1:GFP			AA		
PVX/SlSPL-CNR2:GFP				AAA	
PVX/SlSPL-CNR3:GFP					AAAA
PVX/SlSPL-CNR4:GFP	AAAA				
PVX/SlSPL-CNR5:GFP		AAAAA			
Double-domain mutants					
PVX/SlSPL-CNR12:GFP			AA	AAA	
PVX/SlSPL-CNR13:GFP			AA		AAAA
PVX/SlSPL-CNR14:GFP	AAAA		AA		
PVX/SlSPL-CNR15:GFP		AAAAA	AA		
PVX/SlSPL-CNR23:GFP				AAA	AAAA
PVX/SlSPL-CNR24:GFP	AAAA			AAA	
PVX/SlSPL-CNR25:GFP		AAAAA		AAA	
PVX/SlSPL-CNR34:GFP	AAAA				AAAA
PVX/SlSPL-CNR35:GFP		AAAAA			AAAA
PVX/SlSPL-CNR45:GFP	AAAA	AAAAA			
Triple-domain mutants					
PVX/SlSPL-CNR123:GFP			AA	AAA	AAAA
PVX/SlSPL-CNR124:GFP	AAAA		AA	AAA	
PVX/SlSPL-CNR125:GFP		AAAAA	AA	AAA	
PVX/SlSPL-CNR134:GFP	AAAA		AA		AAAA
PVX/SlSPL-CNR135:GFP		AAAAA	AA		AAAA
PVX/SlSPL-CNR145:GFP	AAAA	AAAAA	AA		
PVX/LeSPL-CNR234:GFP	AAAA			AAA	AAAA
PVX/SlSPL-CNR235:GFP		AAAAA		AAA	AAAA
PVX/SlSPL-CNR245:GFP	AAAA	AAAAA		AAA	
PVX/SlSPL-CNR345:GFP	AAAA	AAAAA			AAAA
Quadri-domain mutants					
PVX/SlSPL-CNR1234:GFP	AAAA		AA	AAA	AAAA
PVX/SlSPL-CNR1235:GFP		AAAAA	AA	AAA	AAAA
PVX/SlSPL-CNR1245:GFP	AAAA	AAAAA	AA	AAA	
PVX/SlSPL-CNR1345:GFP	AAAA	AAAAA	AA		AAAA
PVX/SlSPL-CNR2345:GFP	AAAA	AAAAA		AAA	AAAA
Quint-domain mutants					
PVX/SlSPL-CNR12345:GFP	AAAA	AAAAA	AA	AAA	AAAA

Fig. S2 PVX-based gene expression vectors for characterizing the SlSPL-CNR NLS and VIGC. The five basic amino acid domains are replaced with alanine (A) either individually or in combinations. The genome organization of PVX/GFP and the schematic of SlSPL-CNR are indicated. All mutant proteins are in-framed fused with GFP for expression of relevant fusion proteins.

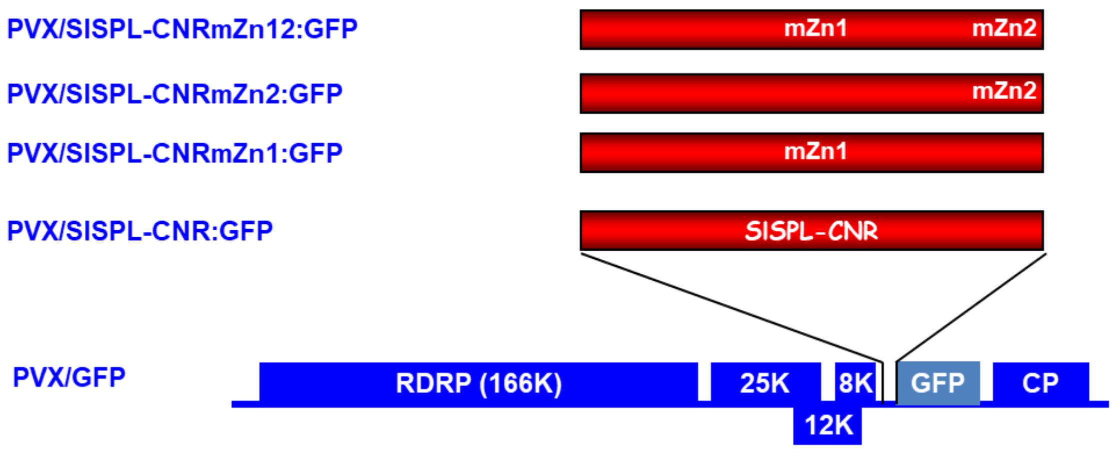

Fig. S3 PVX-based gene expression vectors for characterizing the SlSPL-CNR ZFMs and VIGC. The genome organization of PVX/GFP is indicated. The Zn1, Zn2 and Zn/Zn2 mutated ZFM proteins are in-framed fused with GFP for expression of relevant fusion proteins.

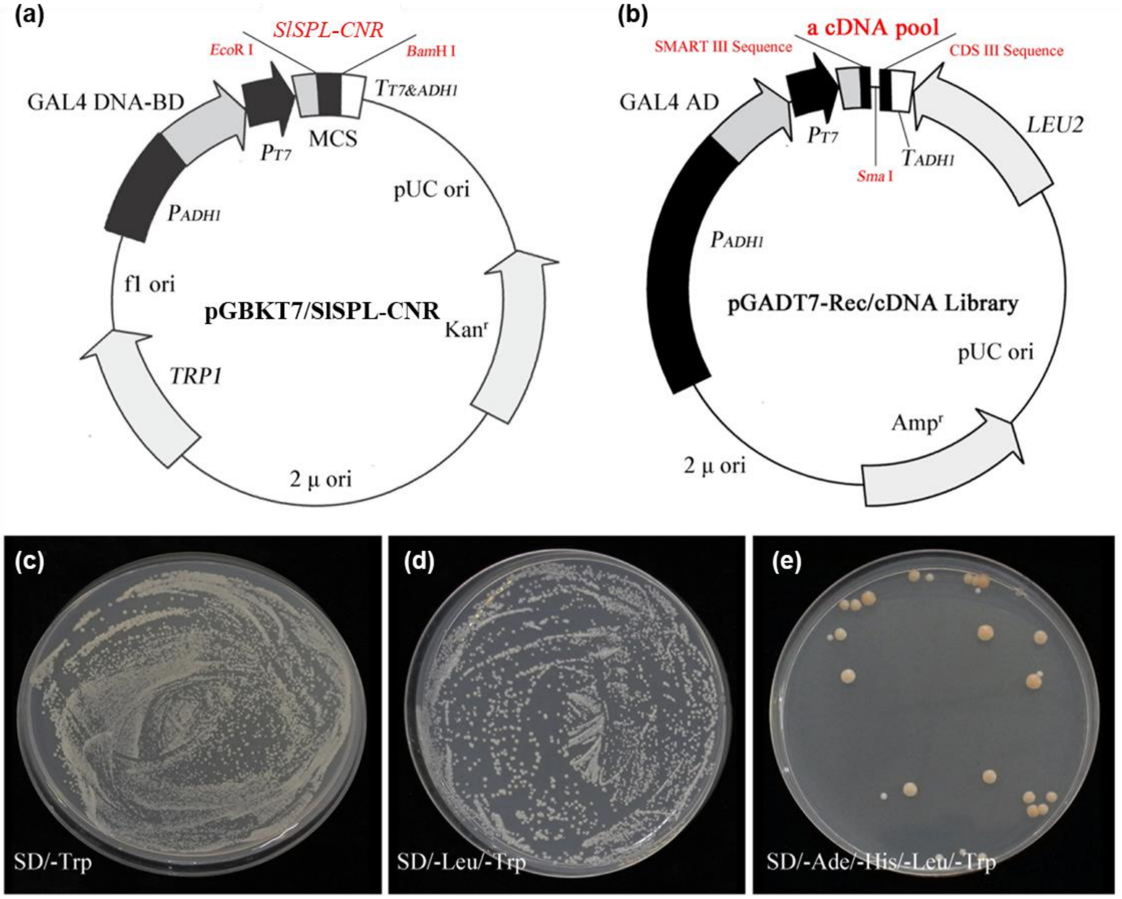

Fig. S4 Yeast-two-hybrid screening of SlSPL-CNR-interacting proteins**.** (a) Bait vector pGBKT7/SlSPL-CNR. (b) Tomato cDNA in prey vector pGADT7/cDNA Library (c-e) Screening on SD/-Trp (c), SD/-Leu/-Trp (d) or SD/-Ade/-His/-Leu/-Trp (e) plates. SD/-Trp: SD medium without tryptophan. Colonies that grow on this medium contain bait plasmid; SD/-Leu/-Trp: SD medium without leucine and tryptophan. Colonies that grow on this medium contain both bait and prey plasmids; SD/-Ade/-His/-Leu/-Trp: SD medium without adenine, histidine, leucine and tryptophan. Colonies that grow on this medium contain both bait and prey plasmids that express bait and prey proteins interacting with each other.

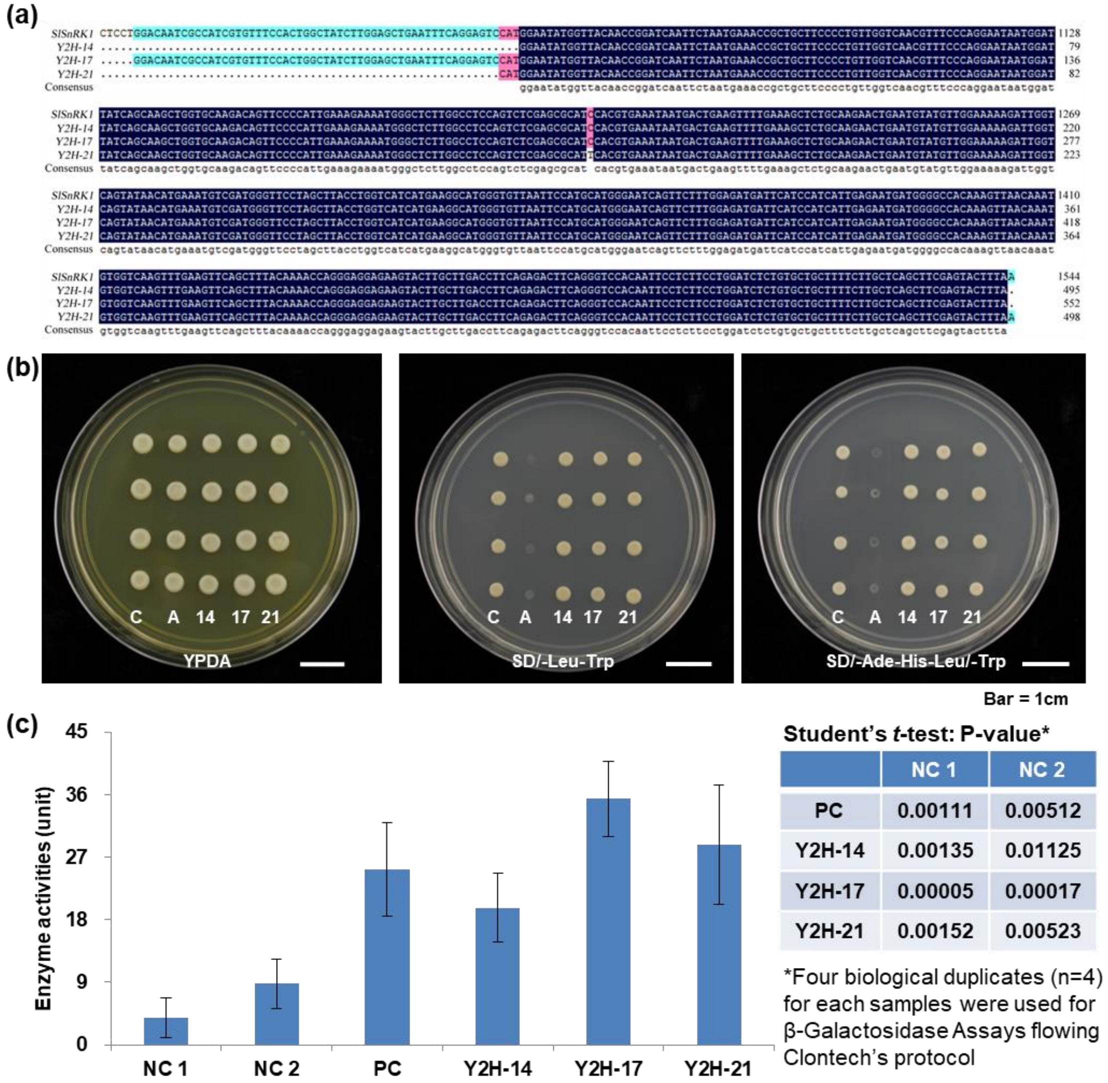

Fig. S5 Yeast-two-hybrid screening identifies three partial sequences coding for *SlSnRK1* polypeptides that interact with SlSPL-CNR. (a) Nucleotide sequence comparisons among *SlSnRK1* (AF143742) and the three identified gene fragments designated Y2H-14, Y2H-17 and Y2H-21. These sequences are all mapped to the 3'-end of the *SlSnRK1* gene. (b) Confirmation of interactions between SlSPL-CNR and the polypeptides encoded by *SlSnRK1* gene fragments Y2H-14 (14), Y2H-17 (17) and Y2H-21 (21), respectively, in Y2H assays. C: positive control, i.e. yeast strain AH109 carrying both pGBKT7-53 and pGADT7-T. A: negative control, i.e. AH109 strain. Bar = 1cm. Yeasts were cultured on YPDA agar plates (YPDA), synthetically defined (SD) medium plate without supplement of leucine (Leu) and tryptophan (Trp; SD/-Leu-Trp), or SD without supplement of adenine (Ade), histidine (His), Leu and Trp (SD/-Ade-His-Leu-Trp). (c) Quantitative

analysis of protein-protein interactions using β-galactosidase activity assay. β-galactosidase assays were performed following Clontech's protocol. 1 unit of β-galactosidase is defined as the amount which hydrolyzes 1 µmol of ONPG (o-nitrophenyl β-D-galactopyranoside) to o-nitrophenol and D-galactose per min per cell. NC1: AH109 strain as Negative control; NC2: negative control 2 (AH109 carrying both pGBKT7 and *SlSPL-CNR* vector as a second negative control); PC: AH109 carrying pGBKT7-53 and pGADT7-T as a positive control; Y2H-14, Y2H-17 and Y2H-21 indicates that AH109 carrying pGBKT7/*SlSPL-CNR* and one of pGADT7/*SlSnRK1* fragment constructs, respectively. Four biological duplicates (n=4) for each sample were used in the β-galactosidase assays (Mean ± S.D). Bars represent standard deviation (S.D.). Student's ***t***-tests were carried out against the two negative controls, respectively. P-values are indicated.

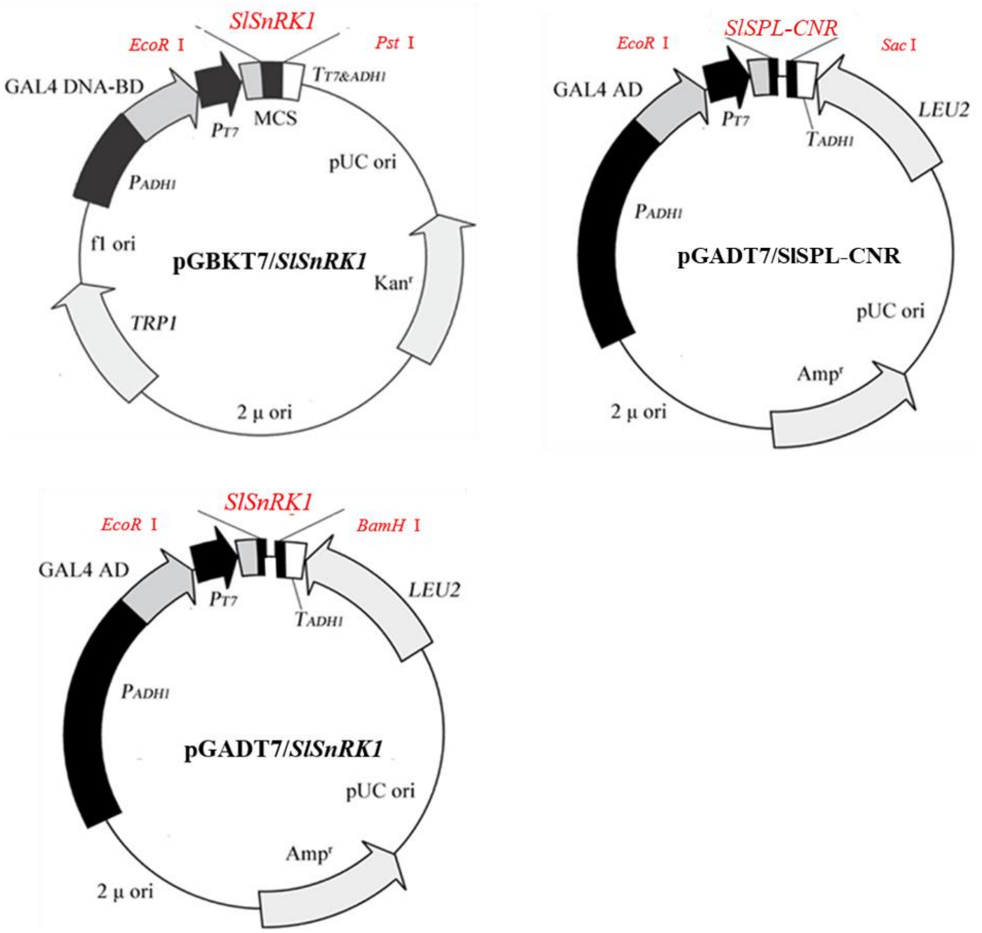

Fig. S6 Construction of SlSnRK1 and SlSPL-CNR full-length gene expression vectors for Y2H protein-protein interaction assay. The full-length *SlSnRK1* gene was cloned in two conformations to produce pGADT7/SlSnRK1 and pGBKT7/SlSnRK1, respectively. In addition to pGBKT7/SlSPL-CNR (Fig. S4a), the full-length SlSPL-CNR gene was also cloned into pGADT7/SlSPL-CNR. The pairs of pGBKT7/SlSPL-CNR and pGADT7/SlSnRK1, pGBKT7/SlSnRK1 and pGADT7/SlSPL-CNR, together with various combinations of vectors were used to verify SlSPL-CNR and SlSnRK1 interactions (Fig. 6).

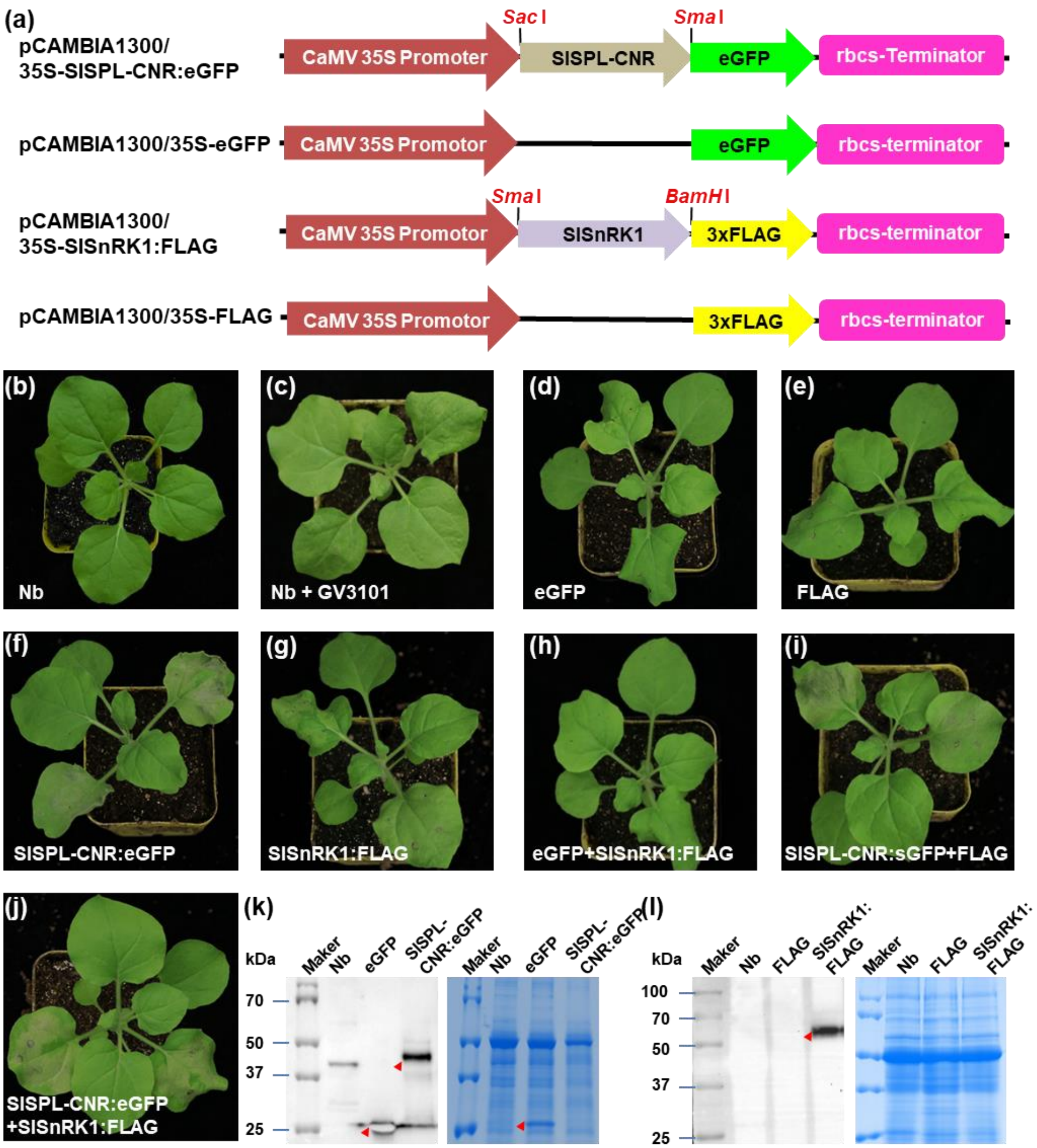

Fig. S7 CoIP analysis of SlSPL-CNR and SlSnRK1 protein interaction. (a) Construction of SlSPL-CNR:eGFP and SlSnRK1:FLAG fusion protein expression cassettes. All constructs were produced in the binary vector pCambia1300. Expression of free or fusion proteins is under the control of CaMV 35S promoter. (b-j) Transient gene expression assays. Young leaves of *Nicotiana benthimiana* (Nb) plants at six-leaf stage were infiltrated or co-infiltrated with *Agrobacterium tumefaciens* GV3101 carrying each of the binary gene expression vectors (a) as indicated in each panel. Nb, non-infiltrated control (b); Nb+GV3101, infiltrated with GV3101 (c); eGFP, infiltrated with

GV3101/pCAMBIA1300/35S-eGFP (d); FLAG, infiltrated with GV3101/pCAMBIA1300/35S-FLAG (e); SlSPL-CNR:eGFP, infiltrated with GV3101/pCAMBIA1300/35S-SlSPL-CNR:eGFP (f); SlSnRK1:FLAG, infiltrated with GV3101/pCAMBIA1300/35S-SlSnRK1:FLAG (g); eGFP+SlSnRK1:FLAG, co-infiltrated with GV3101/pCAMBIA1300/35S-eGFP and GV3101/pCAMBIA1300/35S-SlSnRK1:FLAG (h); SlSPL-CNR:eGFP+FLAG, co-infiltrated with GV3101/pCAMBIA1300/35S-SlSPL-CNR:eGFP and GV3101/pCAMBIA1300/35S-FLAG (i); SlSPL-CNR:eGFP+SlSnRK1:FLAG, co-infiltrated with GV3101/pCAMBIA1300/35S-SlSPL-CNR:eGFP and GV3101/pCAMBIA1300/35S-SlSnRK1:FLAG (j). 3 days after agroinfiltration (DPA), leaves infiltrated with GV3101/pCAMBIA1300/35S-SlSPL-CNR:eGFP alone, or together with GV3101 carrying other gene expression cassettes started to develop necrotic cell death, consistent with results from viral transient expression of SlSPL-CNR. (k,l) Western blot detection of SlSPL-CNR:eGFP and SlSnRK1:FLAG fusion proteins in agroinfiltrated leaf tissues. Total proteins were extracted from control and agroinfiltrated leaves at 3 DPA. Western blots were probed either with antiGFP antibody (k, left panel) or anti3xFLAG antibody (l, left panel). SlSPL-CNR:eGFP and SlSnRK1:FLAG fusion proteins are indicated by red arrow. Free GFP in leaf tissues infiltrated with GV3101/pCAMBIA1300/35S-eGFP were abundant and visible in Coomassie Blue staining gel (red arrow, k, right panel), and become a halo (red arrow) in Western blot detection (k, left panel). Some non-specific detection by the antiGFP antibody was occasionally found in the Nb samples. Equal loading of protein samples were illustrated by a Coomassie Blue staining gels (k, l; right panels). The positions and sizes of protein markers are indicated.

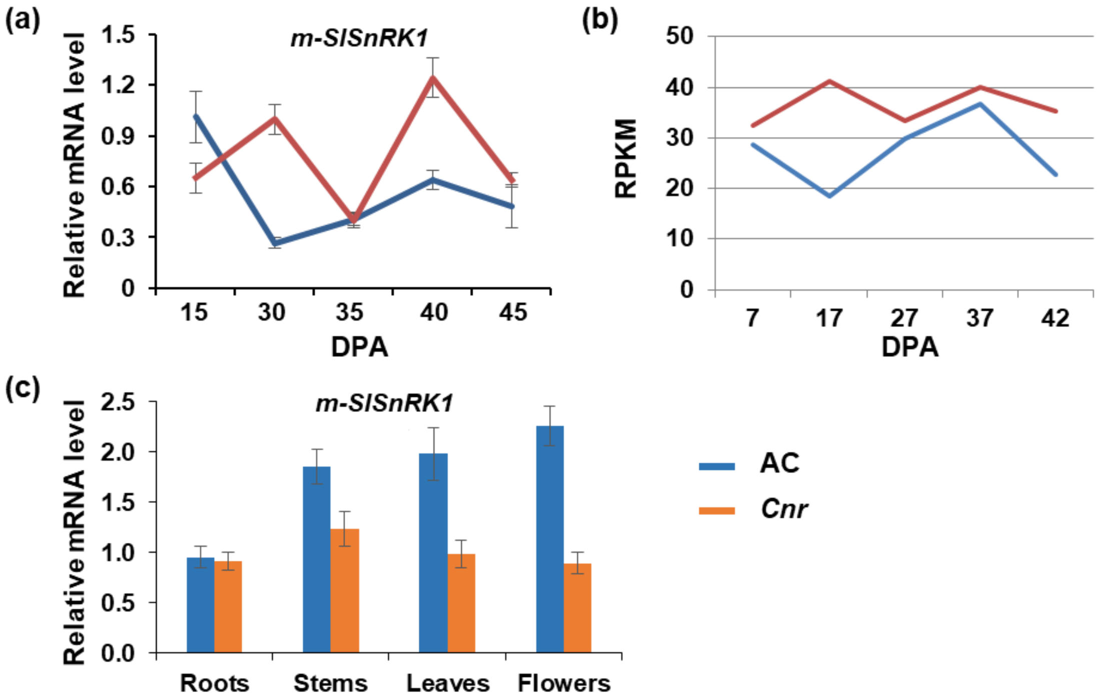

Fig. S8 *SlSnRK1* expression in tomato. (a) qRT-PCR analysis of *SlSnRK1* mRNA level in fruits. DPA: days post-anthesis. (b) RNAseq analysis of *SlSnRK1* mRNA transcripts in fruits. RPKM: Reads Per Kilobase of transcript per Million mapped reads. (c) qRT-PCR analysis of *SlSnRK1* gene expression in different tomato tissues. A set of primers used in (a) and (c) corresponds to the middle portion (*m-SlSnRK1*) of *SlSnRK1* mRNA (Data S1).

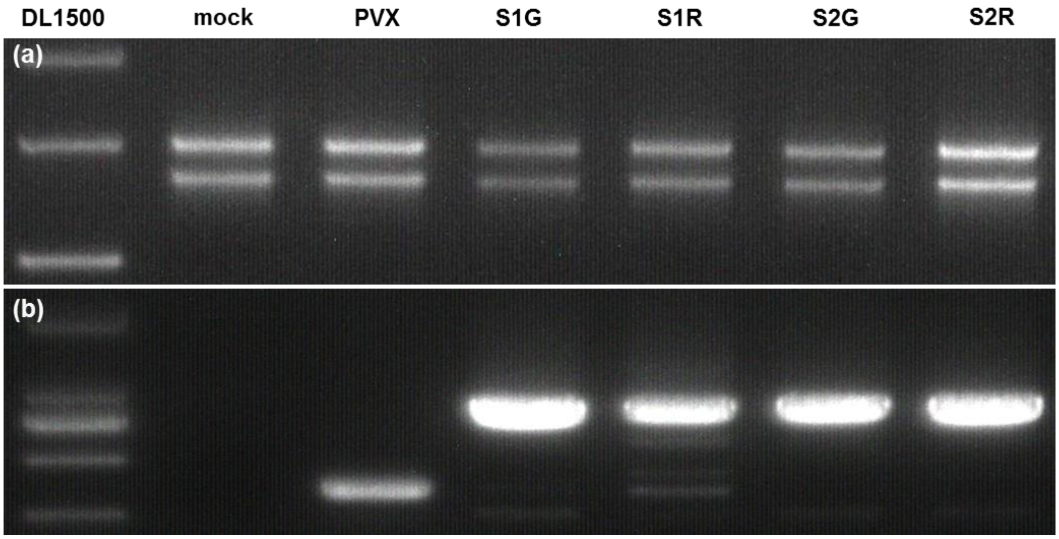

Fig. S9 Detection of PVX/SlSnRK1 in tomato fruits. (a) Gel check of RNA quality. Integrity of 18S and 28S rRNA indicates good quality of RNAs extracted from pericarp tissues from tomato fruits that were mock-injected (mock), or injected with empty PVX VIGS vector (PVX), or PVX/SlSnRK1. (b) RT-PCR detection of PVX or PVX/SlSnRK1 in mock or virus-injected fruits. S1G and S2G indicate that RNAs were extracted from green non-ripe sectors; whilst S1R and S2R from red ripe sectors of two different *SlSnRK1*-silenced fruits.

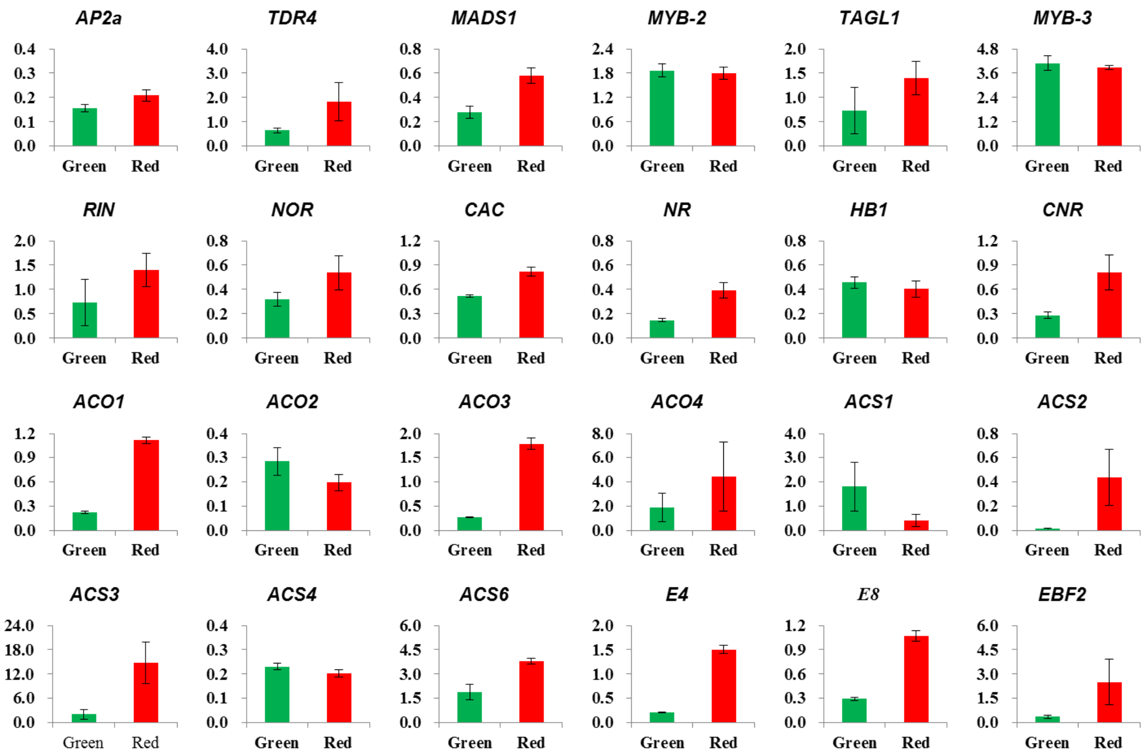

Fig. S10 qRT-PCR analyses of ripening-related TF and ethylene biosynthesis and responsive genes in non-ripe (green bar) and ripe (red bar) of *SlSnRK1*-silenced AC fruits. The relative expression level of each gene was normalized against the 18S rRNA. *AP2a*: *APETAL2a*; *TDR4*: *Agamous-like MADS-box protein AGL8 homolog*; *MADS1*: *MADS-box transcription factor gene1*; *MYB2*: *MYB-box transcription factor gene 2*; *TAGL1*: T*OMATO AGAMOUS-LIKE 1*; *MYB3*: *MYB-box transcription factor gene 3*; *RIN*: *ripening-inhibitor*; *NOR*: *non-ripening*; *CAC*: *clathrin adaptor complex subunit*; *NR*: *never-ripe; HB1*: *homeodomain leucine zipper protein*; *CNR*: *Colorless non-ripening*; *ACO1*: *1-amino-cyclopropane-1-carboxylate oxidase1*; *ACO2*: *1-amino-cyclopropane-1-carboxylate oxidase2*; *ACO3*: *1-amino-cyclopropane-1-carboxylate oxidase3*; *ACO4*: 1-*amino-cyclopropane-1-carboxylate oxidase4*; *ACS1*: *1-amino-cyclopropane-1-carboxylate synthase1*; *ACS2: 1-amino-cyclopropane-1-carboxylate synthase2*; *ACS3*: *1-amino-cyclopropane-1-carboxylate synthase3*; *ACS4*: *1-amino-cyclopropane-1-carboxylate synthase4*; *ACS6*: *1-amino-cyclopropane-1-carboxylate synthase6*; *E4*: *Peptide methionine sulfoxide reductase*; *E8*: *1-aminocyclopropane-1-carboxylate oxidase homolog*; *EBF2*: *ETHYLENE-INSENSITIVE3 binding F-box2*.

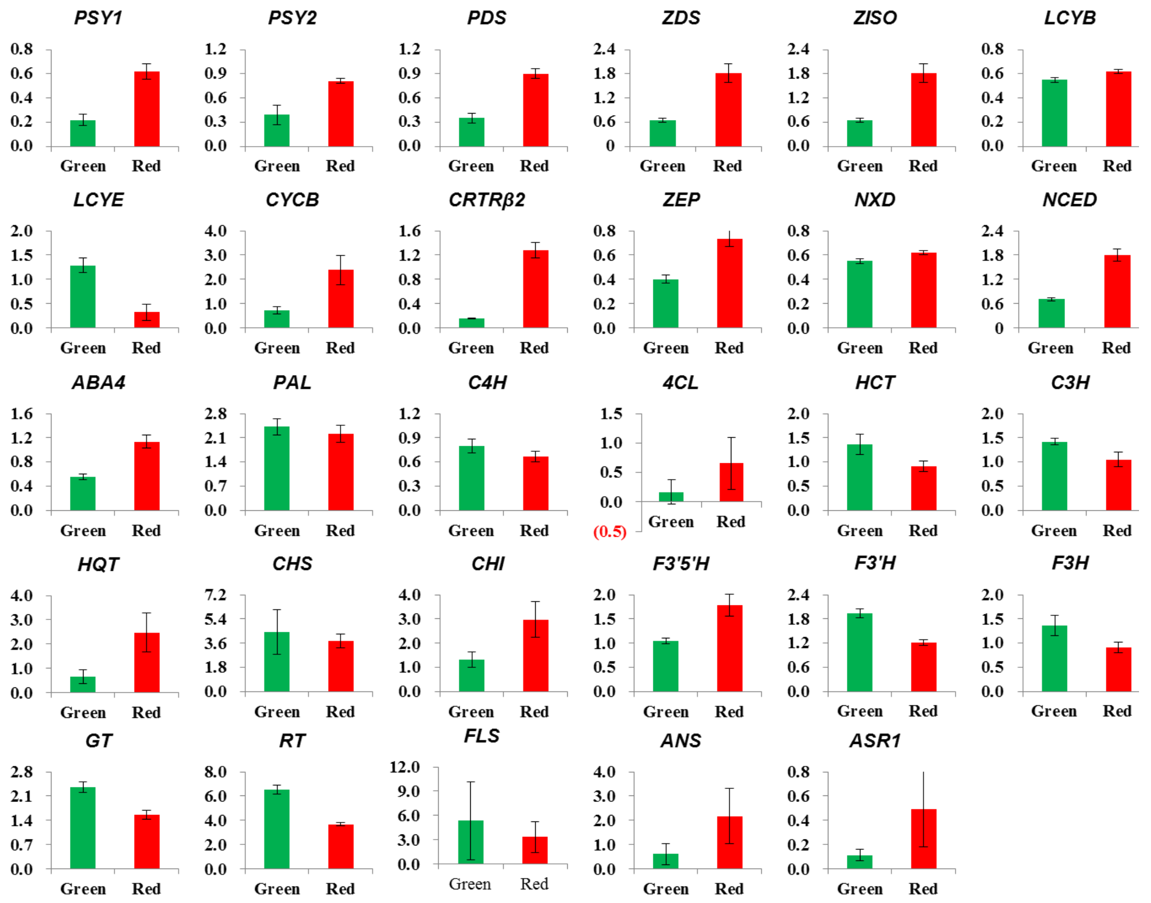

Fig. S11 qRT-PCR analyses of expression of lycopene, ABA, carotenoid and flavonoid biosynthesis and other ripening-related genes in non-ripe (green bar) and ripe (red bar) of *SlSnRK1*-silenced AC fruits. The relative expression level of each gene was normalized against the 18S rRNA. *PSY1: phytoene synthase 1; PSY2*: *phytoene synthase 2*; *PDS*: *phytoene desaturase*; *ZDS*: *zeta-carotene desaturase*; *ZISO*: *zeta-carotene isomerase; LCYB: β-lycopene cyclase*; *LCYE*: *ε-lycopene cyclase*; *CYCB*: *β-lycopene cyclase*; *CRTRβ2*: *β-carotene hydroxylase*; *ZEP*: *zeaxanthin epoxidase*; *NXD*: *neoxanthin synthase*; *NCED*: *9-cis-epoxycarotenoid dioxygenase*; *ABA4*: *abscisic acid responsive4; PAL*: *phenylalanine ammonia lyase*; *C4H*: *cinnamate 4-hydroxylase*; *4CL*: *4-coumaroyl CoA ligase*; *HCT*: *hydroxycinnamoyl transferase*; *C3H*: *p-coumarate 3-hydroxylase; HQT*: *hydroxycinnamoyl-CoA quinate hydroxycinnamoyl transferase*; *CHS*: *chalcone synthase*; *CHI*: *chalcone isomerase*; *F3'5'H*: *flavonoid 3'5'hydroxylase*; *F3'H*: *flavonoid 3'-hydroxylase*; *F3H*: *flavanone 3-hydroxylase*; *GT*: *glucosyltransferase*; *RT*: *rhamnosyltransferase*; *FLS*: *flavonol synthase*; *ANS*: *anthocyanidine synthase*; *ASR1*: *abscisic stress ripening gene 1*.

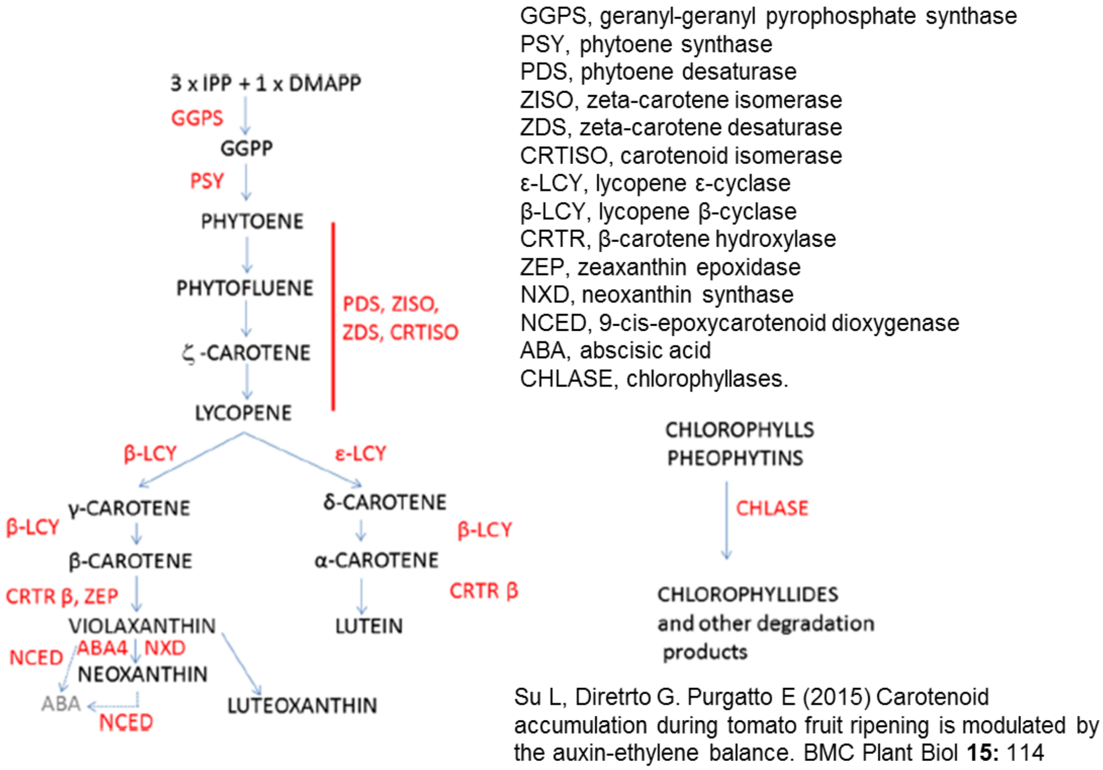

Fig. S12 Genes involved in the lycopene, carotenoid and abscisic acid (ABA) biosynthesis (Fig. S11). Pathways and full gene names as well as their references are indicated.

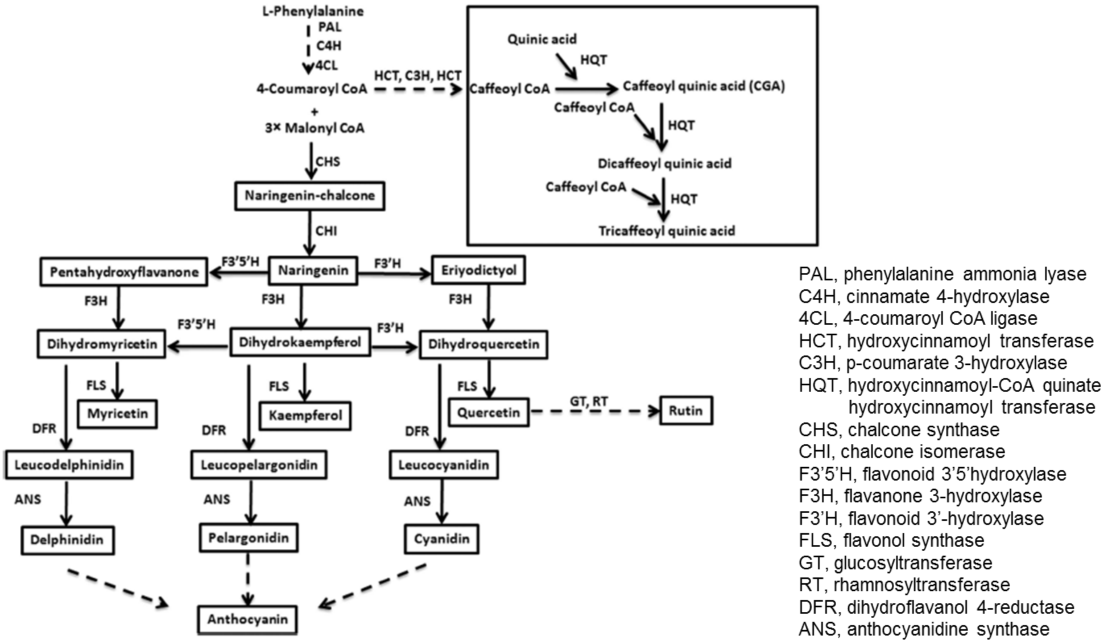

PAL, phenylalanine ammonia lyase
C4H, cinnamate 4-hydroxylase
4CL, 4-coumaroyl CoA ligase
HCT, hydroxycinnamoyl transferase
C3H, p-coumarate 3-hydroxylase
HQT, hydroxycinnamoyl-CoA quinate hydroxycinnamoyl transferase
CHS, chalcone synthase
CHI, chalcone isomerase
F3'5'H, flavonoid 3'5'hydroxylase
F3H, flavanone 3-hydroxylase
F3'H, flavonoid 3'-hydroxylase
FLS, flavonol synthase
GT, glucosyltransferase
RT, rhamnosyltransferase
DFR, dihydroflavanol 4-reductase
ANS, anthocyanidine synthase

Pandey A, Misra P, Choudhary D, Yadav R, Goel R, Bhambhani S, Sanyal I, Trivedi R, Trivedi PK (2015) AtMYB12 expression in tomato leads to large scale differential modulation in transcriptome and flavonoid content in leaf and fruit tissues. Sci Rep 5: 12412.

Fig. S13 Genes involved in the flavonoid biosynthesis (Fig. S11). Pathways and full gene names as well as their references are indicated.

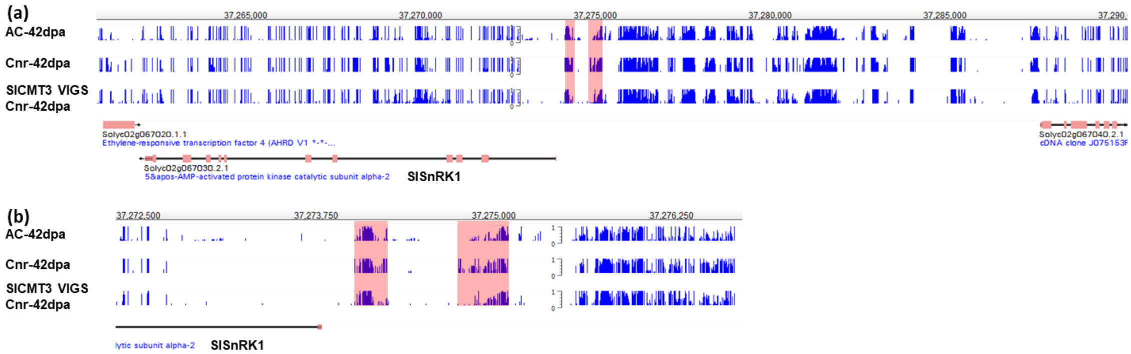

Fig. S14 DNA methylation profiles for the *SlSnRK1* gene. (a, b) Differentially methylated regions (highlighted) in the promoter of *SlSnRK1*. The overall view (a) as well as the enlarged view (b) to show the changed level of DNA methylation. Whole genome bisulfite sequencing data for the wild-type AC and the epimutant *Cnr* pericarps at 42 days post anthesis (dpa) as well as the *SlCMT3*-silenced *Cnr* pericarps at 42 dpa (Zhong *et al*., 2013; Chen *et al*., 2015b) were included for the comparative bioinformatics analysis. Gene ID and its coordinates on the tomato chromosome are indicated.
