# Supplementary material for: Molecular and functional characterization of the SBP-box transcription factor SPL-CNR in tomato fruit ripening and cell death
Source: J Exp Bot. 2020 Feb 4;71(10):2995–3011. doi: 10.1093/jxb/eraa067 (PMC7260717; doi:10.1093/jxb/eraa067)
Supplement: eraa067_suppl_supplementary_tables_S1_S4 [file eraa067_suppl_supplementary_tables_s1_s4.pdf]

Table S1 Summary of cellular localization of wild-type and mutant SlSPL-CNRs and their functionality to induce cell death

**Cellular localization**<br>**Cell Death**	**SlSPL-CNR:GFP fusion protein**
**Cytoplasm**<br>**Non-cell death**	SlSPL-CNR4:GFP, SlSPL-CNR14:GFP, SlSPL-CNR24:GFP,SlSPL-CNR34:GFP, SlSPL-CNR45:GFP, SlSPL-CNR124:GFP, SlSPL-CNR134:GFP, SlSPL-CNR145:GFP, SlSPL-CNR234:GFP, SlSPL-CNR245:GFP, SlSPL-CNR345:GFP, SlSPL-CNR1234:GFP, SlSPL-CNR1245:GFP, SlSPL-CNR1345:GFP, SlSPL-CNR2345:GFP, SlSPL-CNR12345:GFP
**Nucleus**<br>**Cell death**	SlSPL-CNR:GFP, SlSPL-CNR1:GFP, SlSPL-CNR2:GFP, SlSPL-CNR3:GFP, SlSPL-CNR5:GFP, SlSPL-CNR12:GFP, SlSPL-CNR13:GFP, SlSPL-CNR15:GFP, SlSPL-CNR23:GFP, SlSPL-CNR25:GFP, SlSPL-CNR35:GFP, SlSPL-CNR123:GFP, SlSPL-CNR125:GFP, SlSPL-CNR135:GFP, SlSPL-CNR235:GFP, SlSPL-CNR1235:GFP

Table S2 Primers used for constructions of SlSPL-CNR NLS and ZFM mutants

NLS mutants in PVX/GFP	Primers for the first PCR	Templates for the first PCR	Products	Primers for the second PCR	Templates for the second PCR
SlSPL-CNR1:GFP	pp298/pp507: AACGCCTTCGGCAACTAGCAGCAGCATCATCAAAC	PVX/SlSPL-CNR	Frag1a	pp298: CCTCACATCGATGGAAACTAACAAATGGGAAGGGA /pp300: GATGCTCGGCCGGCCCAAATTTTCTCCATGAGAG	Frag1a+Frag1b
	pp300/pp508: TGCTAGTTGCCGAAGGCGTTTGGCAGGTCACAATG		Frag1b		
SlSPL-CNR2:GFP	pp298/pp509: GTGACCTGCCAAAGCAGCTGCGCAACTCCTCTTAG	PVX/SlSPL-CNR	Frag2a		Frag2a+Frag2b
	pp300/pp510: CAGCTGCTTTGGCAGGTCACAATGAGCGCCGCCG		Frag2b		
SlSPL-CNR3:GFP	pp298/pp511: CCCAAATTTTCTCCATGAGAGTCATATGTAATTGCAGCTGCAGCCTCATTGTGACCTG	PVX/SlSPL-CNR-GFP	Frag3a		Frag3a
SlSPL-CNR12:GFP	pp298/pp555: GCAGCTGCGCAACTAGCAGCAGCATCATCAAACTC	PVX/SlSPL-CNRNLS2-GFP	Frag12a		Frag12a+Frag12b
	pp300/pp556: GAGTTTGATGATGCTGCTGCTAGTTGCGCAGCTGC		Frag12b		
SlSPL-CNR13:GFP	pp298/pp511: CCCAAATTTTCTCCATGAGAGTCATATGTAATTGCAGCTGCAGCCTCATTGTGACCTG	PVX/SlSPL-CNRNLS1-GFP	Frag13a		Frag13a
SlSPL-CNRNLS23-GFP	pp298/pp511: CCCAAATTTTCTCCATGAGAGTCATATGTAATTGCAGCTGCAGCCTCATTGTGACCTG	PVX/SlSPL-CNRNLS2-GFP	Frag23a		Frag23a
SlSPL-CNR4:GFP	pp298/pp563: GTCAATACAGCTGCTGCTGCGCTATCCTCTTCAAC	PVX/SlSPL-CNR-GFP	Frag4a		Frag4a+Frag4b
	pp300/pp564: GGATAGCGCAGCAGCAGCTGTATTGACTCTCTCTG		Frag4b		
SlSPL-CNR5:GFP	pp298/pp565: CACACAGCTGCAGCTGCAGCGTATGGCTTGGCATC	PVX/SlSPL-CNR-GFP	Frag5a		Frag5a+Frag5b
	pp300/pp566: TACGCTGCAGCTGCAGCTGTGTGTGAGTTCCATTC		Frag5b		
SlSPL-CNR45:GFP	pp298/pp563: GTCAATACAGCTGCTGCTGCGCTATCCTCTTCAAC	PVX/SlSPL-CNRNLS5-GFP	Frag45a		Frag45a+Frag45b
	pp300/pp564: GGATAGCGCAGCAGCAGCTGTATTGACTCTCTCTG		Frag45b		
SlSP-CNR123:GFP	pp298/pp511: CCCAAATTTTCTCCATGAGAGTCATATGTAATTGCAGCTGCAGCCTCATTGTGACCTG	PVX/SlSPL-CNRNLS12-GFP	Frag123a		Frag123a
SlSPL-CNR1234:GFP	pp298/pp563: GTCAATACAGCTGCTGCTGCGCTATCCTCTTCAAC	PVX/SlSPL-CNRNLS123-GFP	Frag1234a		Frag1234a+Frag1234b
	pp300/pp564: GGATAGCGCAGCAGCAGCTGTATTGACTCTCTCTG		Frag1234b		
SlSPL-CNR1235:GFP	pp298/pp565: CACACAGCTGCAGCTGCAGCGTATGGCTTGGCATC	PVX/SlSPL-CNRNLS123-GFP	Frag1235a		Frag1235a+Frag1235b
	pp300/pp566: TACGCTGCAGCTGCAGCTGTGTGTGAGTTCCATTC		Frag1235b		
SlSPL-CNR12345:GFP	pp298/pp563: GTCAATACAGCTGCTGCTGCGCTATCCTCTTCAAC	PVX/SlSPL-CNRNLS1235-GFP	Frag12345a		Frag12345a+Frag12345b
	pp300/pp564: GGATAGCGCAGCAGCAGCTGTATT		Frag12345b		

	GACTCTCTCTG				
SlSPL-CNR14:GFP	pp298/pp563: GTCAATACAGCTGCTGCTGCGCTATCCTCTTCAAC	PVX/SlSPL-CNRNLS1-GFP	Frag14a		Frag14a+Frag14b
	pp300/pp564: GGATAGCGCAGCAGCAGCTGTATTGACTCTCTCTG		Frag14b		
SlSPL-CNR15:GFP	pp298/pp565: CACACAGCTGCAGCTGCAGCGTATGGCTTGGCATC	PVX/SlSPL-CNRNLS1-GFP	Frag15a		Frag15a+Frag15b
	pp300/pp566: TACGCTGCAGCTGCAGCTGTGTGTGAGTTCCATTC		Frag15b		
SlSPL-CNR24:GFP	pp298/pp563: GTCAATACAGCTGCTGCTGCGCTATCCTCTTCAAC	PVX/SlSPL-CNRNLS2-GFP	Frag24a		Frag24a+Frag24b
	pp300/pp564: GGATAGCGCAGCAGCAGCTGTATTGACTCTCTCTG		Frag24b		
SlSPL-CNR25:GFP	pp298/pp565: CACACAGCTGCAGCTGCAGCGTATGGCTTGGCATC	PVX/SlSPL-CNRNLS2-GFP	Frag25a		Frag25a+Frag25b
	pp300/pp566: TACGCTGCAGCTGCAGCTGTGTGTGAGTTCCATTC		Frag25b		
SlSPL-CNR34:GFP	pp298/pp563: GTCAATACAGCTGCTGCTGCGCTATCCTCTTCAAC	PVX/SlSPL-CNRNLS3-GFP	Frag34a		Frag34a+Frag34b
	pp300/pp564: GGATAGCGCAGCAGCAGCTGTATTGACTCTCTCTG		Frag34b		
SlSPL-CNR35:GFP	pp298/pp565: CACACAGCTGCAGCTGCAGCGTATGGCTTGGCATC	PVX/SlSPL-CNRNLS3-GFP	Frag35a		Frag35a+Frag35b
	pp300/pp566: TACGCTGCAGCTGCAGCTGTGTGTGAGTTCCATTC		Frag35b		
SlSPL-CNR124:GFP	pp298/pp563: GTCAATACAGCTGCTGCTGCGCTATCCTCTTCAAC	PVX/SlSPL-CNRNLS12-GFP	Frag124a		Frag124a+Frag124b
	pp300/pp564: GGATAGCGCAGCAGCAGCTGTATTGACTCTCTCTG		Frag124b		
SlSPL-CNR125:GFP	pp298/pp565: CACACAGCTGCAGCTGCAGCGTATGGCTTGGCATC	PVX/SlSPL-CNRNLS12-GFP	Frag125a		Frag125a+Frag125b
	pp300/pp566: TACGCTGCAGCTGCAGCTGTGTGTGAGTTCCATTC		Frag125b		
SlSPL-CNR134:GFP	pp298/pp563: GTCAATACAGCTGCTGCTGCGCTATCCTCTTCAAC	PVX/SlSPL-CNRNLS13-GFP	Frag134a		Frag134a+Frag134b
	pp300/pp564: GGATAGCGCAGCAGCAGCTGTATTGACTCTCTCTG		Frag134b		
SlSPL-CNR135:GFP	pp298/pp565: CACACAGCTGCAGCTGCAGCGTATGGCTTGGCATC	PVX/SlSPL-CNRNLS13-GFP	Frag135a		Frag135a+Frag135b
	pp300/pp566: TACGCTGCAGCTGCAGCTGTGTGTGAGTTCCATTC		Frag135b		
SlSPL-CNR234:GFP	pp298/pp563: GTCAATACAGCTGCTGCTGCGCTATCCTCTTCAAC	PVX-CNRNLS23-GFP	Frag234a		Frag234a+Frag234b
	pp300/pp564: GGATAGCGCAGCAGCAGCTGTATTGACTCTCTCTG		Frag234b		
SlSPL-CNR235:GFP	pp298/pp565: CACACAGCTGCAGCTGCAGCGTATGGCTTGGCATC	PVX/SlSPL-CNRNLS23-GFP	Frag235a		Frag235a+Frag235b
	pp300/pp566: TACGCTGCAGCTGCAGCTGTGTGT		Frag23b		

	GAGTTCCATTC				
SlSPL-CNR145:GFP	pp298/pp563: GTCAATACAGCTGCTGCTGCGCTATCCTCTTCAAC	PVX/SlSPL-CNRNLS15-GFP	Frag145a		Frag145a+Frag145b
	pp300/pp564: GGATAGCGCAGCAGCAGCTGTATTGACTCTCTCTG		Frag145b		
SlSPL-CNR245:GFP	pp298/pp563: GTCAATACAGCTGCTGCTGCGCTATCCTCTTCAAC	PVX/SlSPL-CNRNLS25-GFP	Frag245a		Frag245a+Frag245b
	pp300/pp564: GGATAGCGCAGCAGCAGCTGTATTGACTCTCTCTG		Frag245b		
SlSPL-CNR345:GFP	pp298/pp563: GTCAATACAGCTGCTGCTGCGCTATCCTCTTCAAC	PVX/SlSPL-CNRNLS35-GFP	Frag345a		Frag345a+Frag345b
	pp300/pp564: GGATAGCGCAGCAGCAGCTGTATTGACTCTCTCTG		Frag345b		
SlSPL-CNR1245:GFP	pp298/pp563: GTCAATACAGCTGCTGCTGCGCTATCCTCTTCAAC	PVX/SlSPL-CNRNLS125-GFP	Frag1245a		Frag1245a+Frag1245b
	pp300/pp564: GGATAGCGCAGCAGCAGCTGTATTGACTCTCTCTG		Frag1245b		
SlSPL-CNR1345:GFP	pp298/pp563: GTCAATACAGCTGCTGCTGCGCTATCCTCTTCAAC	PVX/SlSPL-CNRNLS135-GFP	Frag1345a		Frag1345a+Frag1345b
	pp300/pp564: GGATAGCGCAGCAGCAGCTGTATTGACTCTCTCTG		Frag1344b		
SlSPL-CNR2345:GFP	pp298/pp563: GTCAATACAGCTGCTGCTGCGCTATCCTCTTCAAC	PVX/SlSPL-CNRNLS235-GFP	Frag2345a		Frag2345a+Frag2345b
	pp300/pp564: GGATAGCGCAGCAGCAGCTGTATTGACTCTCTCTG		Frag2345b		

ZFM mutants

PVX/SlSPL-CNRmZn1:GFP

PP503: 5'-GGTATGGCTTGGCATCTGCCATATCTGCAGTAGCCTGATCGACCTGAGCAGAAGGATGTG-3'

PP504: 5'-GGCAGATGCCAAGCCATACCATCGCCGCCACAAGGTGGCTGAGTTCGCTTCAAAGTCTCC-3'

PP503/PP504：For Zn1 mutant. Combining with primer pp298, change Cys^{52} to Ala^{52} (nt214) and Cys^{57} to Ala^{57} (229) by the first PCR amplification, then annealing with the PCR product amplified by the second PCR using pp300 & pp504. Finally primers pp298 and pp300 were used to amplify the whole length SlSPL-CNR with mutants of Cys^{52} to Ala^{52}, Cys^{57} to Ala^{57}, Cys^{74} to Ala^{74}, His^{77} to Ala^{77}.

PVX/SlSPL-CNRmZn2:GFP

PP505: 5'- CAAACTCTGCTAACAGAGCAAATCTGCTAGCTTGCTGAGCGAATCGCTTCTGG-3'

PP506: 5'-TGCTCTGTTAGCAGAGTTTGATGATGCTAAGAGGAGTGCTCGAAGGCGTTTGG-3

PP505/PP506：For Zn2 mutant. Combining with primer pp298, change Cys^{93} to Ala^{93} (nt394), Cys^{96} to Ala^{96} (nt346) and His^{100} to Ala^{100} (nt358) by the first PCR amplification, then annealing with the PCR product amplified by the second PCR using pp300 & pp506. Finally primers pp298 and pp300 were used to amplify the whole length SlSPL-CNR with mutants of Cys^{93} to Ala^{93}, Cys^{96} to Ala^{96}, His^{100} to Ala^{100} and Cys^{112} to Ala^{112}.

PVX/SlSPL-CNRmZn12:GFP

The Zn1/Zn2 double mutant was constructed as that for PVX/SlSPL-CNRmZn2:GFP, but using PVX/SlSPL-CNRmZn1:GFP plasmid DNA as temples for the first and second PCR reactions.

Table S3 Primers used for qRT-PCR

Name	Sequence (5' – 3')
Q-18S-F	CGGCTACCACATCCAAGGAAGG
Q-18S-R	GAGCTGGAATTACCGCGGCTG
**Genes related to ethylene biosynthesis**	
Q-ACS1-F	GTGCTTCAAACAAAGGGAC
Q-ACS1-R	GTCCTAACCAAAGGCGAAT
Q-ACS2-F	GTAGGTGTTGAGAAAAGTGGAG
Q-ACS2-R	GTCTTAACGAACTAATGGTGAGG
Q-ACS3-F	CATCTCTGAAAATCAGAAGAGGCT
Q-ACS3-R	CCATAAGTCCATTTCAGCGTCA
Q-ACS4-F	AAATCTCCACCTTCACTAACGAAC
Q-ACS4-R	CCTAAGTCCTTGGAAAGACTAGACAC
Q-ACS6-F	CAATACTGTAGAACAAGGAGC
Q-ACS6-R	GGTACTCAGTGAAATAGTCGAC
Q-ACO1-F	CACTAACGGGAAGTACAAGAG
Q-E4-F	AGGGTAACAACAGCAGTAGCA
Q-E4-R	CCCAACCTCCGTCTTCAC
Q-E8-F	GGCACCATTCAACATACCG
Q-E8-R	CTTTCACCGAAGAAGCACG
Q-EBF2-F	TTACCAGGTGTGTGGAAGG
Q-EBF2-R	CCGACATTAGTAATACCACGA
Q-ACO1-R	CTGCATCACTTCCTGGATTGTA
Q-ACO2-F	CAACTCCTCAAAGACGGTCG
Q-ACO2-R	GTCCCGTCTTTTTGTGCGAT
Q-ACO3-F	ATGGGACTCGGATGTCACTAGC
Q-ACO3-R	CTTCCATAGCCTTCATTGCTTC
Q-ACO4-F	CTGTCAACTTAGGTCCAATA
Q-ACO4-R	GCTCACTACCAAACAACAG
**Transcription factor genes relate to fruit ripening**	
*Q-*Sl*SPL-CNR-F*	GCTCTCTATCTTCTGTCAATTCCCG
*Q-*Sl*SPL-CNR-R*	TCGAATACTAGCAGACAGTGCCAAC

Q-AP2a-F	GGAGTATGAATCCGATGAAGGT
Q-AP2a-R	CGATTCCAAATTGTGGTCTT
Q-LeHB1-F	CTACGACGAGCAGTCACCG
Q-LeHB1-R	GGAACCATACAGCCACCT
Q-MADS1-F	GTGTAGCTGGATTTCCACTTCG
Q-MADS1-R	GCCGCTGCATTCACCTCAT
Q-MYB-2F	GGGAAGCCTTAGTTCAGATAGTGAT
Q-MYB-2R	CTATTCCCTACATTATTGCTCGTT
Q-MYB-3F	TGGCAAGATAATGATGAGTTAGTATGG
Q-MYB-3R	AAAGCCAAGATACAATGGTACTATG
Q-CAC-F	CCTCCGTTGTGATGTAACTGG
Q-CAC-R	ATTGGTGGAAAGTAACATCATCG
Q-NR-F	ATCAGGTTGCTGTCGCTCTT
Q-NR-R	GGCCATCTCTGCTTCTTGTC
Q-NOR-F	AGAGAACGATGCATGGAGGTTTGT
Q-NOR-R	ACTGGCTCAGGAAATTGGCAATGG
Q-TAGL1-F	GCAATAACTCCCTGCCTGTA
Q-TAGL1-R	AGATGAAGAGCCTTGACCC
Q-TDR4-F	ACTGGACTCTCCTCACCTTGGGG
Q-TDR4-R	AGCTGCACCTTGCTGCTGTGA
Q-MADS-RIN-F	AAGGAACCCAAACTTCATCAG
Q-MADS-RIN-R	TTGTCCCAAATCCTCACCTA

Carotenoid biosynthetic pathway

Reference

Su L., Diretrto G., Purgatto E., 2015. Carotenoid accumulation during tomato fruit ripening is modulated by the auxin-ethylene balance. BMC Plant Biology 15, 114

Q-PSY1-F	AGAGGTGGTGGAAAGCAA
Q-PSY1-R	TCTCGGGAGTCATTAGCAT
Q-PSY2-F	GAGGTGACAAGGTAACATAAAGG
Q-PSY2-R	CCCACAACAAAGCAACAGAC
Q-PDS-F	GGTCACAAACCGATACTGCT
Q-PDS-R	AAACCAGTCTCGTACCAATCTC
Q-ZDS-F	AGTGGTTTCTGTCTAAAGGTGG

Q-ZDS-R	ACCGAGCACTCATGTTATCAC
Q-ZISO-F	CCTTCTTCTTCCTATACCCGTCG
Q-ZISO-R	AGCGTGTGAGCTAAGCACCA
Q-CRTRβ-F	CTTCTTTCCTACGGTTTCTTCCA
Q-CRTRβ-R	CTCTTATGAACCAGTCCATCGT
Q-ZEP-F	ATGATAGACCGCCAACCTTTAGTT
Q-ZEP-R	CCATGCATCCCCCTTGAC
Q-NCED-F	CATGAACTTGAACACCCTTTGC
Q-NCED-R	CGTTTCGAACGTAAACGCCT
Q-ABA4-F	TGTGCTCGGACTTCTGTACG
Q-ABA4-R	ACTTGCCTTGCAGCAAAAAG
Q-NXD-F	CGATGAGCTTGTGGTGATTG
Q-NXC-R	CTTTCCGGTTTCTGTGGAAG
Q-LCYβ-F	TTGACTTAGAACCTCGTTATTGG
Q-LCYβ-R	AACAGTTCCCTTTGTCATTATCTC
Q-LCYε-F	GCCACAGGTTATTCAGTCGTCA
Q-LCYε-R	CCAGTCCAAATAGGAAAAACGAT
Q-CYCB-F	CGACGTGATCATTATCGGAGC
Q-CYCB-R	GTGGTGAAGGGTCAACACAACA
**Flavonoid biosynthetic pathway**<br>Reference<br>Pandey A., Misra P., Choudhary D. et al., 2015. AtMYB12 expression in tomato leads to large scale differential modulation in transcriptome and flavonoid content in leaf and fruit tissues. Scientific Reports 5, 12412.	
Q-PAL-F	AACCTATCTCGTGGCTCTTT
Q-PAL-R	TCTTTTTCGCTGAATCTTGC
Q-C4H-F	CAACAGAAAGGAGAGATCAACGAG
Q-C4H-R	CACAGCCTGAAGGTATGGAAGC
Q-4CL-F	ACACACAAAGGCTTAGTCACGA
Q-4CL-R	AACAGAGGCAACACACACATCA
Q-CHS-F	TGGTCACCGTGGAGGAGTATC
Q-CHS-R	GATCGTAGCTGGACCCTCTGC
Q-CHI-F	GTTTTTCACAAACCAACAGTTCTGAT

Q-CHI-R	GAAGCAGTGCTCGATTCCATAAT
Q-F3H-F	CACACCGATCCAGGAACCAT
Q-F3H-R	GCCCACCAACTTGGTCTTGTA
Q-F3’H-F	GCACCACGAATGCACTTGC
Q-F3’H-R	CGTTAGTACCGTCGGCGAAT
Q-F3’5’H-F	GGCAATTGGACGAGATCCTG
Q-F3’5’H-R	AAGGAACCTCTCGGGAGTGAA
Q-FLS-F	GAGCATGAAGTTGGGCCAAT
Q-FLS-R	TGGTGGGTTGGCCTCATTAA
Q-ANS-F	GAACTAGCACTTGGCGTCGAA
Q-ANS-R	TTGCAAGCCAGGCACCATA
Q-GT-F	CGAACGACGAAACACTGTTGA
Q-GT-R	TGCAGCATAGATGGCATTGG
Q-RT-F	CTGGCAATGCAAACAGAGTGA
Q-RT-R	TCGACTTGCGGAAGAGTGAGA
Q-C3H-F	CATAAACTCTACCACCGTCTCC
Q-C3H-R	AATCCATCCCATTTCTACTCAA
Q-HCT-F	AGGTGAAAAACTCAACGATGGT
Q-HCT-R	ACACTAGGCGTGTGGAAATTAG
Q-HQT-F	GTGTTTTGTTTGTTGAGGCTGA
Q-HQT-R	TGATGAAGTGGATGGATGAGAG
Q-ASR1-F	CCTGTTCCACCACAAGGACAA
Q-ASR1-R	GTGCCAAGTTTACCGATTTGC

Table S4 Additional primers and their use for construction of gene expression cassettes

**Constructs/primers**	**Sequence (5' – 3')**
**pGBK(AD)T7/*SlSnRK1*-F**	GAGTCGGAATTCATGGACGGAACAGCAGTGCAGGGCA (*Eco*RI)
**pGBKT7/*SlSnRK1*-R**	GAGTCGCTGCAGTTAAAGTACTCGAAGCTGAGCAAGA (*Pst*I)
**pGADT7/*SlSnRK1*-R**	TCGACAGGATCCTTAAAGTACTCGAAGCTGAGCAAGA (*BamHI*)
**pGADT7/*SlSPL-CNR*-F**	TCGACACCCGGGTATGGAAACTAACAAATGGGAAG (*Sma*I)
**pGADT7/*SlSPL-CNR*-R**	ACTCCAGAGCTCGTCAGCCCAAATTTTCTCCATG (*Sac*I)
**pGBKT7/*SlSPL-CNR*-F**	GAGTCGGAATTCATGGAAACTAACAAATGGGAAGGG (*Eco*RI)
**pGBKT7/*SlSPL-CNR*-R**	TCGACAGGATCCTCAGCCCAAATTTTCTCCATGAGAG (*Bam*HI)
**pCAMBIA1300/35s-SlSnRK1-3×FLAG-F**	TCGACACCCGGGATGGACGGAACAGCAGTGCAGGGCA (*SmaI*)
**pCAMBIA1300/35s-SlSnRK1-3×FLAG-R**	TCGACAGGATCCAAGTACTCGAAGCTGAGCAAGA (*BamHI*)
**pCAMBIA1300/35s-SlSPL-CNR-eGFP-F**	ACTCCAGAGCTCATGGAAACTAACAAATGGGAAGGGA (*Sac*I)
**pCAMBIA1300/35s-SlSPL-CNR-eGFP-R**	TCGACACCCGGGGCCCAAATTTTCTCCATGAGAG (*Sma*I)
**pCAMBIA1300/35s-eGFP-Seq-F**	TGTCACTTTATTGTGAAGATAGTG
**pCAMBIA1300/35s-eGFP-Seq-R**	AGATGAACTTCAGGGTCAGC
**pCAMBIA1300/35s-3×FLAG-Seq-F**	TAAAGGAAAGGCCATCGTTG
**pCAMBIA1300/35s-3×FLAG-Seq-R**	ACTAGTGGATCATTTGTCATCA
