# Supplementary material for: Molecular and functional characterization of the SBP-box transcription factor SPL-CNR in tomato fruit ripening and cell death
Source: J Exp Bot. 2020 Feb 4;71(10):2995–3011. doi: 10.1093/jxb/eraa067 (PMC7260717; doi:10.1093/jxb/eraa067)
Supplement: eraa067_suppl_supplementary_dataset_S1 [file eraa067_suppl_supplementary_dataset_s1.pdf]

Data S1 Background information about *SlSnRK1*

(A) Primers used for construction of the *SlSnRK1* VIGS vector PVX/SlSnRK1 (Fig. 8A)

SlSnRK1-F: ACTCCA**CGGCCG**TAGGACGGAACAGCAGTGCAG (*Eag*I site underlined)

SlSnRK1-R: GAGTGC**TCCGGA**CGCATGATATTGCTCAAACC (*Bsp*EI site underlined)

(B) *Solanum lycopersicum SlSnRK1* mRNA [Solyc02g067030.2.1; NCBI Reference: AF143742 (Bradford *et al.*, 2003); NM_001317176.1 (Avila *et al.*, 2012)]

```
1    aggggggaaa aattaacaga atcaaaaatc agtgaactcc attcttgtgc ttctgcagcc
61   atccttaccc ccacccccg aatctcctcc accccacgac tggcctcaac ttcttccccc
121  tgtattccag gaggccattg atactctctt tcatcaccaa atgttgaaga taatcgcttg
181  attttcccaa ttttcatatt ttgttgttga atttcttcaa tctgcttctt ggggtttagc
241  ttctgccagg aaaatggacg gaacagcagt gcagggcacc agcagtgttg actcattttt
301  acggaactat aaactcggga aaacacttgg cattggatcg ttcggcaaag ttaaaatagc
361  tgaacatacg ttaacagggc acaaagttgc tgtcaagatt cttaatcgtc gaaaaatcag
421  gaatatggac atggaggaga aagtccgtag agaaatcaaa atattgagat tgttcatgca
481  tcctcatatt atacggcttt atgaggtcat agagacacca tcagatatat atgttgtgat
541  ggagtatgtg aaatctggcg agttatttga ttacattgtt gagaagggca gattgcagga
601  ggatgaagct cgtaactttt ttcagcagat aatttctggt gtggagtact gccatagaaa
661  catggtggtt catagagacc ttaagcctga aaacctcctt ctggactcca aatggaatgt
721  gaagatcgca gattttggtt tgagcaatat catgcgcgat ggtcattttc tgaagacaag
781  ttgcggaagc ccaaactatg ctgccccaga ggttatatca ggtaaattgt atgctggccc
841  tgaggtagat gtatggagct gtggtgttat tctttatgct cttctctgtg gcacccttcc
901  gtttgacgat gaaaacatac ccaatctttt taagaaaata aagggtggaa tatatactct
961  gcccagccat ttatcagctg gtgcgaggga tttgattccg aggatgctta tagtcgaccc
1021 aatgaagcga atgactattc ctgagattcg cctgcaccct tggttccaag ctcatttgcc
1081 acgctatttg gccgtgcctc caccagatac aacccaacaa gcaaagaaga tcgatgaaga
1141 gattcttcaa gaggtggtta agatgggatt tgacaggaac aaccttactg agtctcttcg
1201 caatagagtt caaaatgagg gcactgttgc atactatctg ctcctggaca atcgccatcg
1261 tgtttccact ggctatcttg gagctgaatt tcaggagtcc atggaatatg gttacaaccg
1321 gatcaattct aatgaaaccg ctgcttcccc tgttggtcaa cgtttcccag gaataatgga
1381 ttatcagcaa gctggtgcaa gacagttccc cattgaaaga aaatgggctc ttggcctcca
1441 gtctcgagcg catccacgtg aaataatgac tgaagttttg aaagctctgc aagaactgaa
1501 tgtatgttgg aaaaagattg gtcagtataa catgaaatgt cgatgggttc ctagcttacc
1561 tggtcatcat gaaggcatgg gtgttaattc catgcatggg aatcagttct ttggagatga
1621 ttcatccatc attgagaatg atggggccac aaagttaaca aatgtggtca agtttgaagt
1681 tcagctttac aaaaccaggg aggagaagta cttgcttgac cttcagagac ttcagggtcc
1741 acaattcctc ttcctggatc tctgtgctgc ttttcttgct cagcttcgag tactttaaag
1801 tctccgaaat aaggagctaa gttggaaaaa gcccatgctt gtatataatt ggtataccag
1861 ctcagttact gcattttgtc ttgttaacaa attccaccct gcttggtcag aggtgcctag
1921 caactctttt tttcttttga ttcgctagga gatctagctc actctctttt ttaacgttta
1981 tggaatttca gttacctaaa aaaaaaa
```

5'-SlSnRK1: qRT-PCR target (Fig. 8F)
Q-F1: CAGGAGGCCATTGATACTCT
Q-R1: GAGTCAACACTGCTGGTGCC
Region: 128-294 bp (underlined)
Product Length: 167 bp

A fragment that covers the sequence highlighted red was amplified by RT-PCR and cloned into the VIGS vector PVX/SlSNF1 (Fig. 8A). The start codon AUG was replaced with a stop codon UAG to ensure no SlSnRK1 polypeptide could be produced from PVX/SlSnRK1.

M-SlSnRK1: qRT-PCR target(Fig. 8F)
Q-F2: CTGCCCCAGAGGTTATATCA
Q-R2: TGGCTGGGCAGAGTATATAT
Region: 801-969 bp (underlined)
Product Length: 169 bp

SlSnRK1 coding sequence: highlighted yellow

3'-SlSnRK1: qRT-PCR target (Fig. 8F)
Q-F3: GAAGTACTTGCTTGACCTTC
Q-R3: GCAGTAACTGAGCTGGTATAC
Region: 1705-1872 bp (underlined)
Product Length: 168 bp

Bradford KJ, Downie AB, Gee OH, Alvarado V, Yang H, Dahal P. 2003. Abscisic acid and gibberellin differentially regulate expression of genes of the SNF1-related kinase complex in tomato seeds. Plant Physiology **132,** 1560-1576.

Avila J, Gregory OG, Su D, Deeter TA, Chen S, Silva-Sanchez C, Xu S, Martin GB, Devarenne TP. 2012. The beta-subunit of the SnRK1 complex is phosphorylated by the plant cell death suppressor Adi3. Plant Physiology **159,** 1277-1290.
